# Author Correction: SGK1 inhibition in glia ameliorates pathologies and symptoms in Parkinson disease animal models

**DOI:** 10.1038/s44321-025-00270-y

**Published:** 2025-08-11

**Authors:** Oh-Chan Kwon, Jae-Jin Song, Yunseon Yang, Seong-Hoon Kim, Ji Young Kim, Min-Jong Seok, Inhwa Hwang, Je-Wook Yu, Jenisha Karmacharya, Han-Joo Maeng, Jiyoung Kim, Eek-hoon Jho, Seung Yeon Ko, Hyeon Son, Mi-Yoon Chang, Sang-Hun Lee

**Affiliations:** 1 Department of Biochemistry and Molecular Biology, College of Medicine, Hanyang University, Seoul, Korea; 2 Hanyang Biomedical Research Institute, Hanyang University, Seoul, Korea; 3 Graduate School of Biomedical Science and Engineering, Hanyang University, Seoul, Korea; 4 Korea Department of Microbiology and Immunology, Institute for Immunology and Immunological Diseases, Brain Korea 21 PLUS Project for Medical Science, Yonsei University College of Medicine, Seoul, South Korea; 5 College of Pharmacy, Gachon University, Incheon, 21936 Korea; 6 Department of Life Science, University of Seoul, Seoul, 02504 Republic of Korea

## Abstract

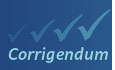

**Correction to:** *EMBO Molecular Medicine* (2021) 13:e13076. 10.15252/emmm.202013076 | Published online 1 March 2021

The authors contacted the journal after being made aware of an image reuse within their paper. The authors were able to locate the correct images. After reviewing the corrected images and source data provided by the authors, the journal retracts and replaces the following figures.

**Figure 7T is retracted and replaced**

**Figure 7:**
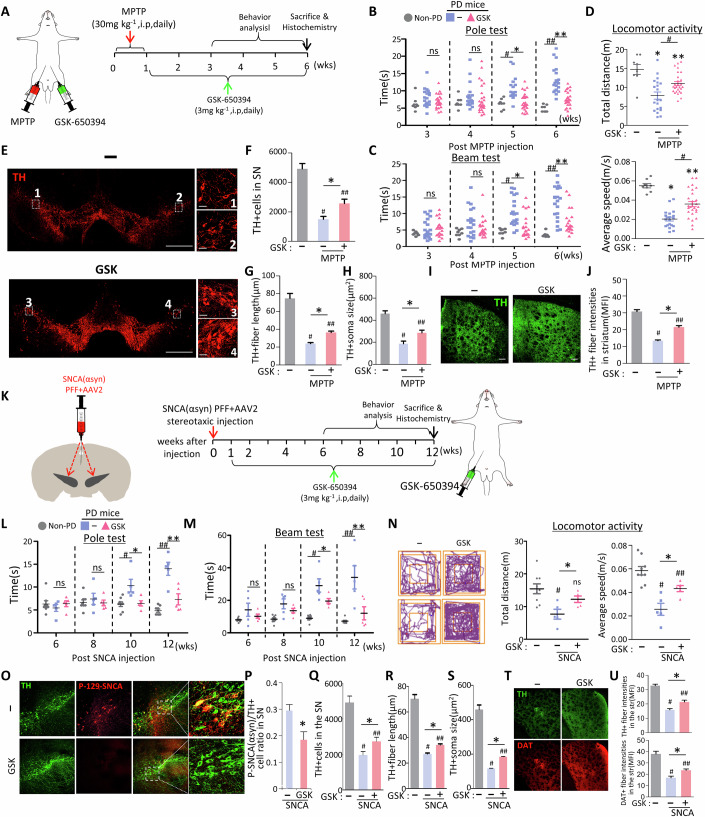
Original.

**Figure 7:**
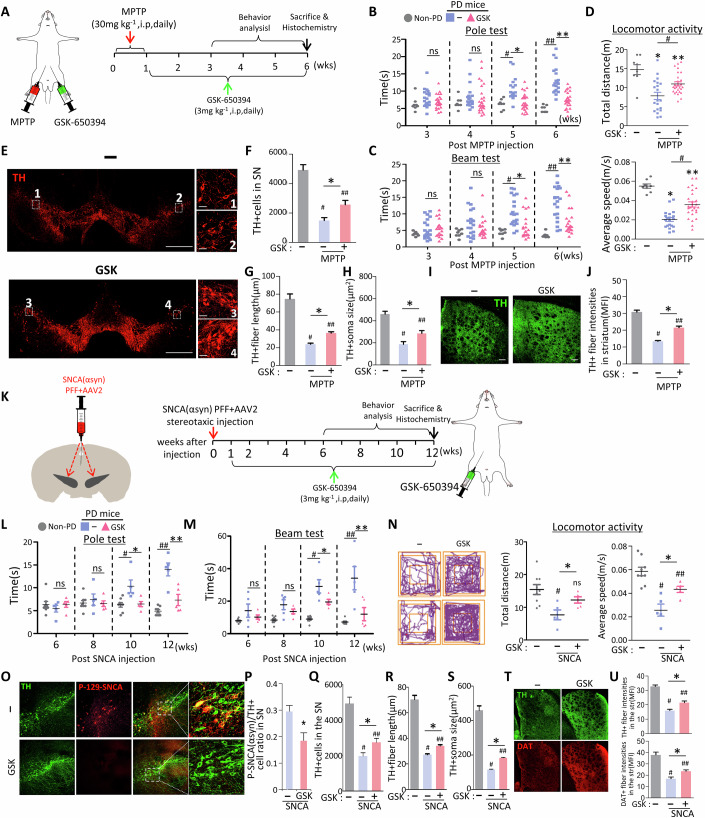
Corrected. Source data are available online for this figure.

**Figure 9D is retracted and replaced**.

**Figure 9:**
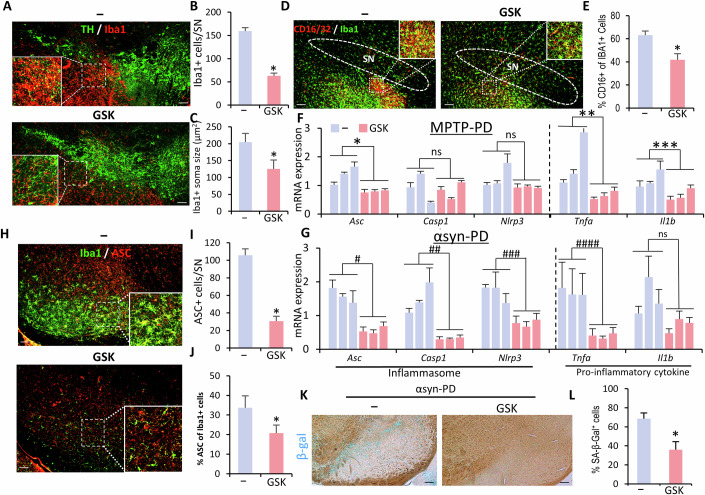
Orignal.

**Figure 9:**
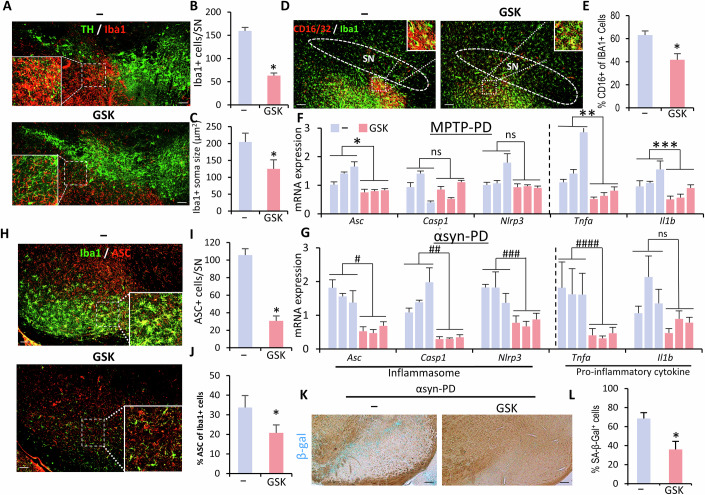
Corrected. Source data are available online for this figure.

**Corresponding source data is published with this correction**.

Author statement:

In Fig. 7T of the originally published article, the image of the striatum from the control mouse was identical to the one in Fig. 8N. This was an error. We have corrected it by replacing the duplicate image with the correct one.

In Fig. 9D of the originally published article, an incorrect region was inadvertently included in the inset due to a labeling error. This issue has been resolved by preparing a revised figure with the proper inset.

This correction does not affect the conclusions of the manuscript.

All authors agree to this author correction.

## Supplementary information

Source Data for corrected figures

