# Supplementary material for: SGK1 inhibition in glia ameliorates pathologies and symptoms in Parkinson disease animal models
Source: EMBO Mol Med. 2021 Mar 1;13(4):e13076. doi: 10.15252/emmm.202013076 (PMC8033538; doi:10.15252/emmm.202013076)
Supplement: Supplementary file 1 — Appendix [file EMMM-13-e13076-s002.pdf]

Table of contents

Appendix Figure legends

Appendix Figures

Appendix Tables

Appendix Figure legends

Appendix Figure S1. RNA-seq analyses for the genes commonly regulated by SGK1 inhibition andNurr1+Foxa2 (N+F) overexpression in cultured glia. A and B, Venn-diagram summarizing the overlapof the genes downregulated (A) and upregulated (B) from the glial culture treated with the SGK1inhibitor GSK-650394 vs. the untreated control (left circle) and the culture transduced withNurr1+Foxa2 vs. the mock-transduced control (right circle). The numbers of genes are indicatedin the Venn-diagram. C–F, GO and KEGG analyses for the genes commonly down- (726 genes,C and E) or upregulated (777 genes, D and F). Red bars indicate p values, and negative logs ofthe p values are plotted on the X-axis.

Appendix Figure S2. SGK1 inhibition upregulates expression and activity of glutamate transporters in cultured glial cells. A and B, Expression of glutamate transporters in RNA-seq and q-PCR analyses. C, Glutamate uptake activity. n=3. ANOVA. Significantly different at p=0.00001#, 0.0018##, 0.0393* (GLT1), 0.002#, 0.0007## (GLAST) in graph B and p=0.0026#, 0.0001##, 0.012* in graph C.

Appendix Figure S3. The combined α-syn overexpression + PFF treatment to mDA neuronal cultures induces the formation of α-syn aggregates detected by immunoblot for SNCA(α-syn) (A) and immunocytochemical analyses against phosphorylated α-syn at serine 129 (p129-α-syn) (B). n=3, student's t-test. Significantly different at p=0.016*, 0.0006**, 0.0005***, 0.0319**** in graph A and p= 0.0054* in graph B. Scale bar, 20μm.

Appendix Figure S4. Effect of the SGK1 inhibitor GSK-650394 treatment on α-syn monomer and aggregate levels in primary mDA neuron cultures. Seven days after the initial α-syn PFF treatment, the neuronal cultures were exposed to GSK-650394 for 4 days. The levels of α-syn monomers and aggregates were detected by immunoblot for SNCA(α-syn) (A) and immunocytochemical analyses against p129-α-syn (B). n=3. ANOVA. Significantly different at p=0.0003#, 0.0003## (monomer), 0.0002#, 0.0007## (dimer), 9.03E-06#, 7.77E-06## (trimer), 1.04E-06#, 1.16E-06## (oligomer) in graph A and p=0.0016*, 0.0022** in graph B. ns, not significant, Scale bar, 20μm.

Appendix Figure S5. SGK1 inhibition in VM-glia potentiates their ability to rescue neuronal cells from mitochondrial and oxidative stress. VM-derived mDA neuron cultures were treated with glial CM (GCM) prepared in VM-glial cultures transduced with sh-Sgk1 (or sh-control as a control) in the presence of H_2O_2 (150 μM, 2hr). Intra-neuronal mitochondrial and oxidative stress was estimated by Mito-timer (A), Mito-Sox (B), DCF-staining (C), and reduced glutathione (GSH) levels (D). ANOVA. n=3. Significantly different at p=1.18E-15#, 9.72E-08##, 0.011*, 0.048**, 1.98E-10+ in graph A and p=1.98E-24#, 4.99E-15##, 1.43E-08*, 0.000003**, 1.80E-17+ in graph B and 1.37E-24#, 1.09E-12##, 0.000037###, 1.58E-10*, 0.0002**, 5.36E-18+ in graph C. Significantly different at p=0.0329#, 0.0054##, 0.0383* in graph D. Scale bar, 20μm.

Appendix Figure S6. Effect of the SGK1 inhibitor GSK-650394 on neuronal viability in primary mDA neuron cultures. A, Endogenous SGK1 expression in the primary cultures of mDA neurons, astrocytes, and microglia. The cultures were derived from mouse VM at E15 (mDA neuron) and post-natal day 5 (astrocyte, microglia). Significantly different at p=0.00002*, 0.009** in graph A. B, Immunocytochemical analysis to determine mDA neuron degeneration. The pure mDA neuron cultures were treated with GSK-650394 (or DMSO as control). Two days after the inhibitor treatment, mDA neuronal viability and degeneration (induced by H_2O_2, 250μM, 3hr) were assessed using TuJ1+ (TH+) neuron counts and neurite outgrowths. n=3. Scale bar, 20μm.

Appendix Figure S7. SGK1 inhibition in cultured astrocytes and microglia potentiates their individual and interactive neuroprotective actions in a paracrine manner. A, Schematic presentation of the experimental procedures and groups. On the right, the experimental groups for CM treatments are summarized with numbers and abbreviations. Astrocytes and microglia derived from the VMs of mouse pups were cultured and transduced with the lentivirus expressing sh-Sgk1 (or sh-cont as the control). Medium was conditioned in the cultures of astrocytes (ACM) and microglia (MCM) and directly administered to mDA neuron cultures. To assess the interactive neurotrophic actions between astrocytes and microglia, ACM was administered to cultured microglia for 2 days, and then the microglial CM challenged by ACM

(A-MCM) was collected and administered to cultured mDA neurons. Similarly, astrocyte CM challenged by MCM (M-ACM) was prepared. Cell viability and mDA neuronal resistance to H2O2-mediated toxic insult (250 μM, 4hr) were determined using the CCK8 assay (B,C) and MAP2+ neuronal (D,E) and TH+ mDA neuronal (F,G) count. ANOVA. n=3 cultures. Significantly different at p=3.036E-12#, 1.053E-09##, 1.003E-10###, 3.073E-10####, 9.382E-09####, 0.0058*, 0.0018**, 0.0472&, 0.0347@ in graph B and p=1.088E-12#, 0.029*, 0.036**,0.0178***, 0.0154**** in graph C. Significantly different at p=6.951E-11#, 4.146E-09##, 3.08E-10###, 4.112E-08####, 2.273E-10#####, 0.0081**, 0.0143+, 0.0393@ in graph D and p=8.145E-13#, 0.0345*, 0.0042***, 0.0309**** in graph E. Significantly different at p=1.414E-12#, 0.00001##, 3.297E-08###, 0.00001####, 8.266E-07#####, 0.0482+ in graph F and 1.058E-15#, 0.0489*, 0.041*** in graph G.

Appendix Figure S8. SGK1 expression upregulated in the SN of α-syn-PD model mice. SGK1 expression levels were estimated in the midbrain SN of normal control (-) and PD (+) mice treated with the combined α-syn-lentivirus+PFF (1 month after α-syn injection). A and B, total SGK1 expression levels estimated by real-time PCR (A) and IHC (B). Significantly different at p=0.001* in graph A and p=1.4E-06* in graph B. C, Cell type-specific SGK1 expressions assessed in neuron (MAP2a+), astrocyte (GFAP+), and microglia (IBA1+). SGK1 positive cells were indicated by arrow heads. Significantly different at p=0.039*, 0.013**, 0.038*** in graph C. Student's t-test. n=3 (A), 3 (control) and 5 (PD) mice (B and C). Scale bar, 20μm.

Appendix Figure S9. Schematic summary for the action mechanisms of glial SGK1 inhibition to ameliorate PD pathologies.

Appendix Figure S1

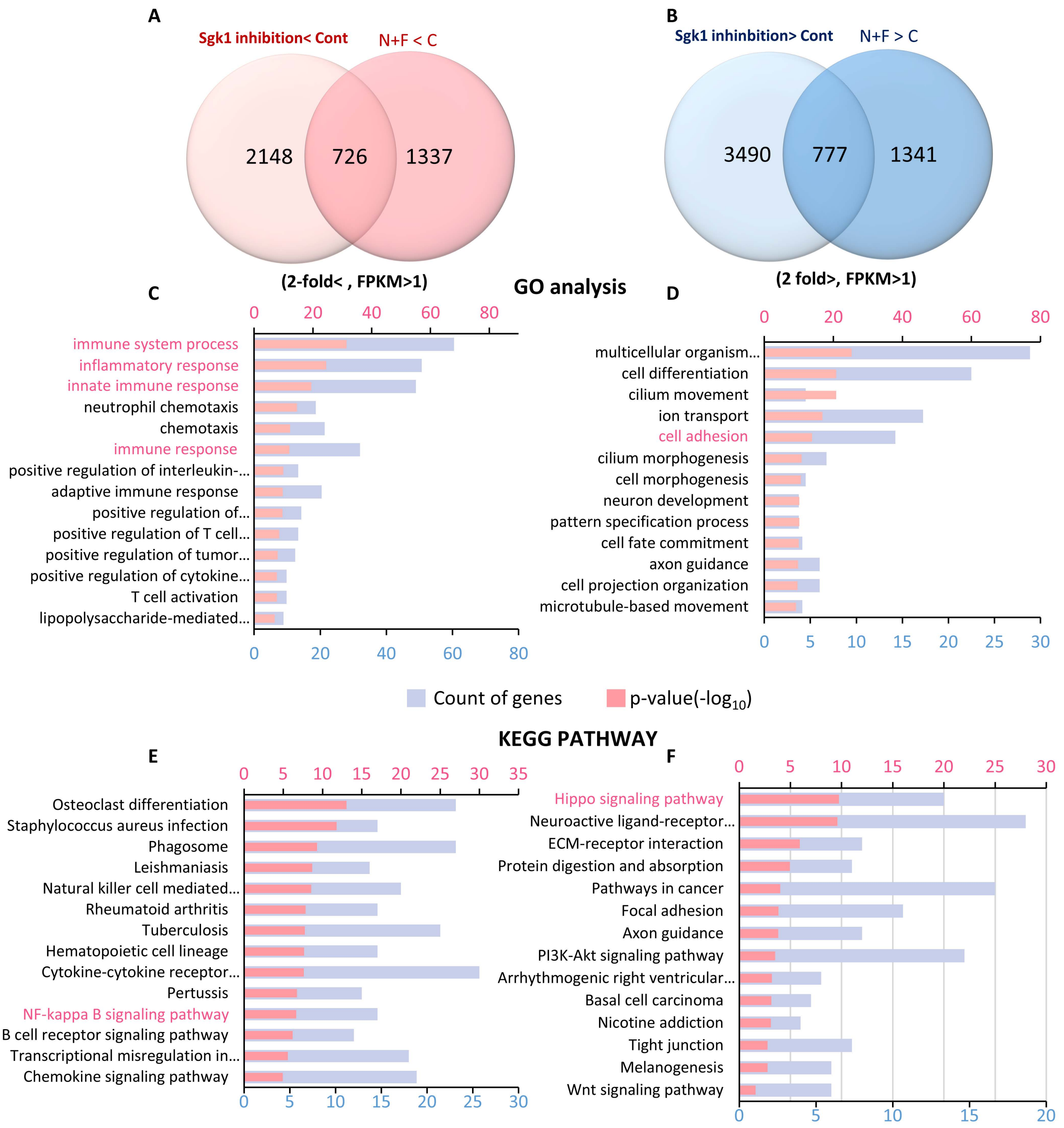

Appendix Figure S2

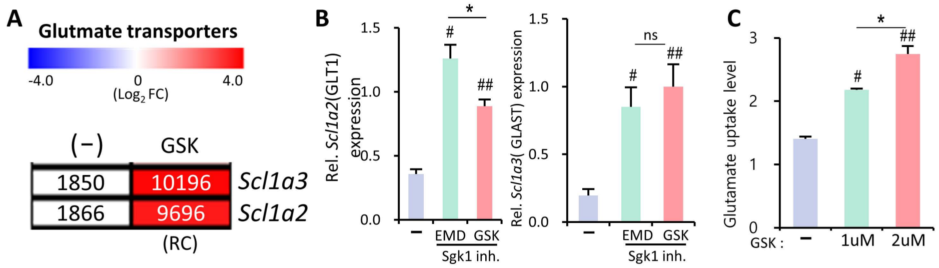

Appendix Figure S3

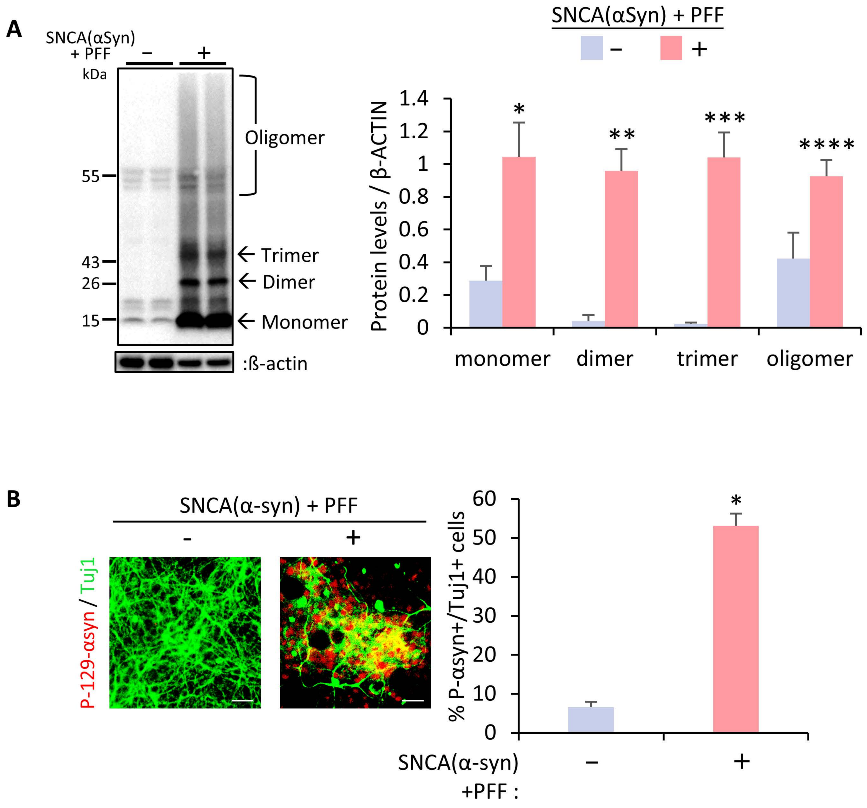

Appendix Figure S4

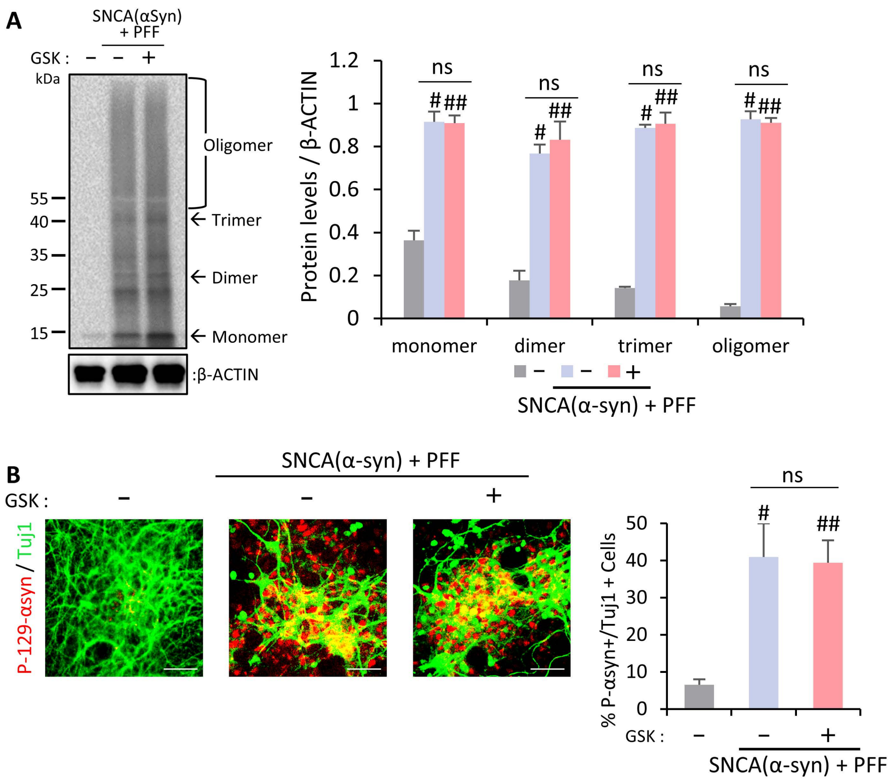

Appendix Figure S5

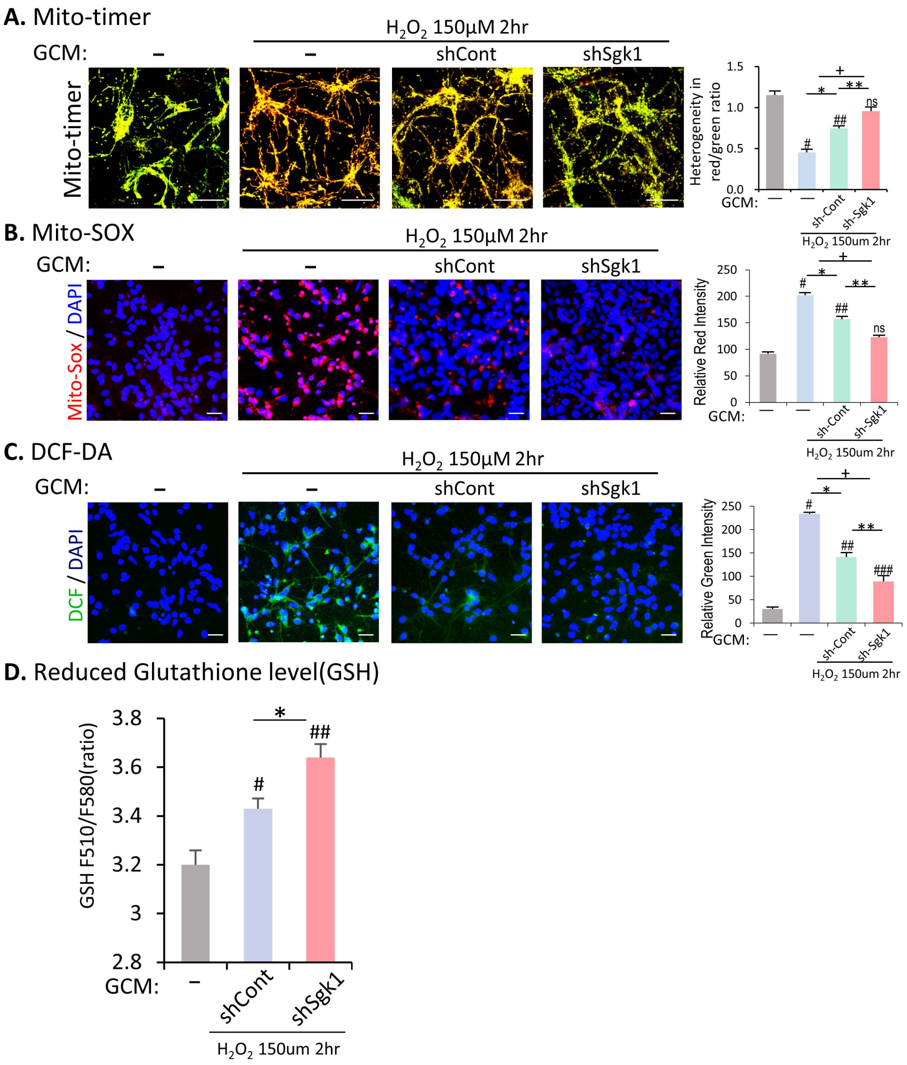

Appendix Figure S6

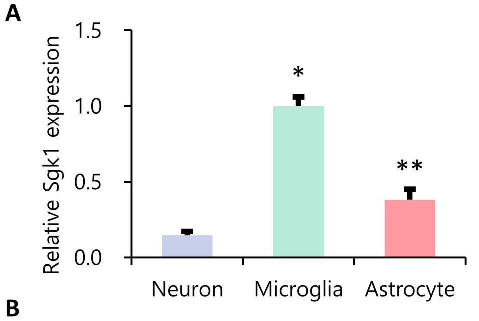

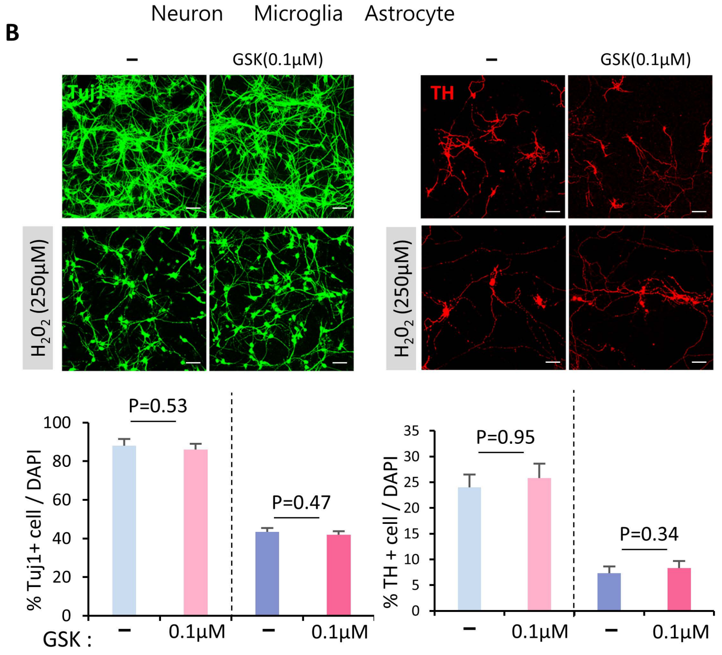

Appendix Figure S7

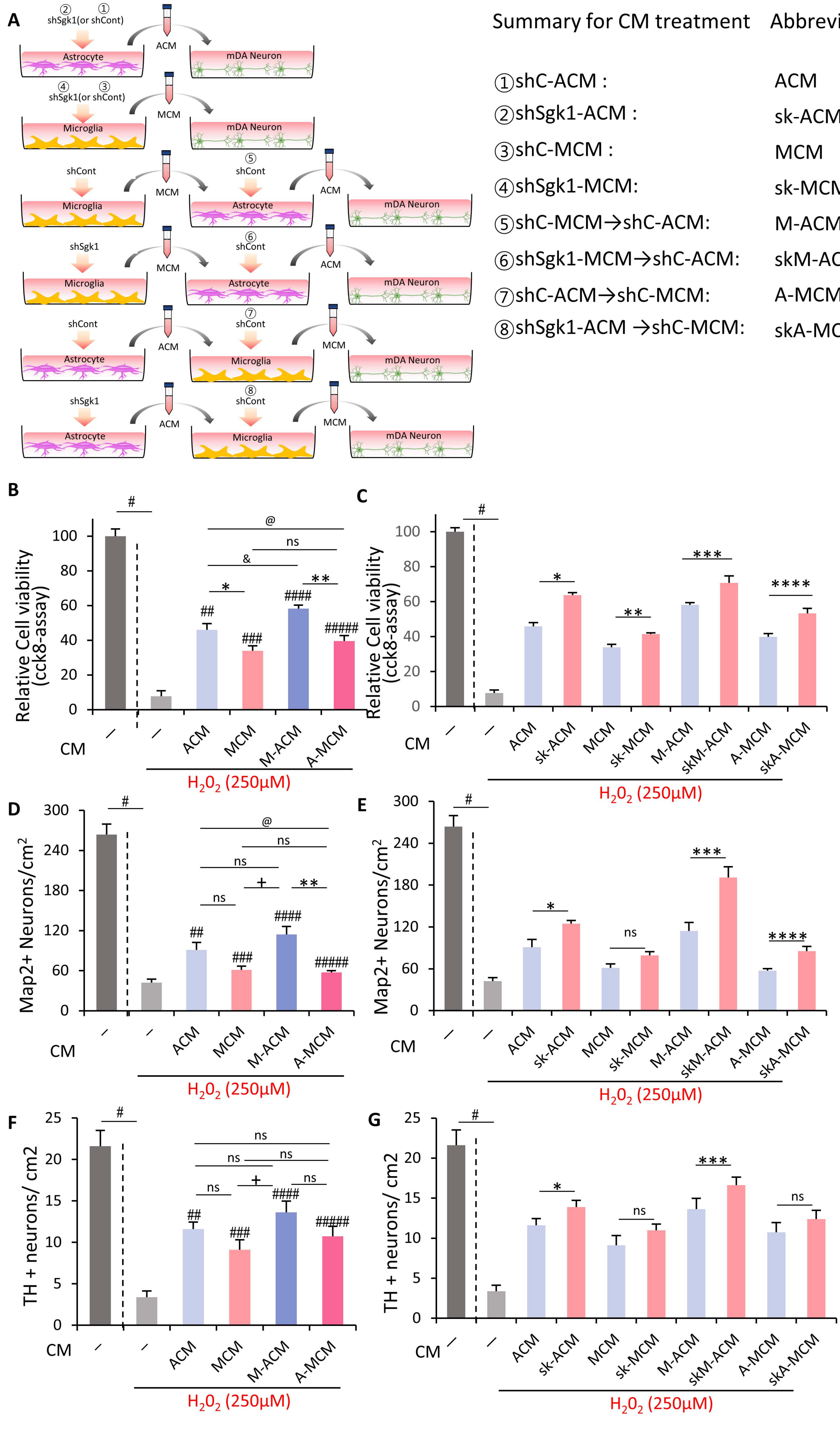

Summary for CM treatment	Abbreviation
①shC-ACM :	ACM
②shSgk1-ACM :	sk-ACM
③shC-MCM :	MCM
④shSgk1-MCM:	sk-MCM
⑤shC-MCM→shC-ACM:	M-ACM
⑥shSgk1-MCM→shC-ACM:	skM-ACM
⑦shC-ACM→shC-MCM:	A-MCM
⑧shSgk1-ACM →shC-MCM:	skA-MCM

Appendix Figure S8

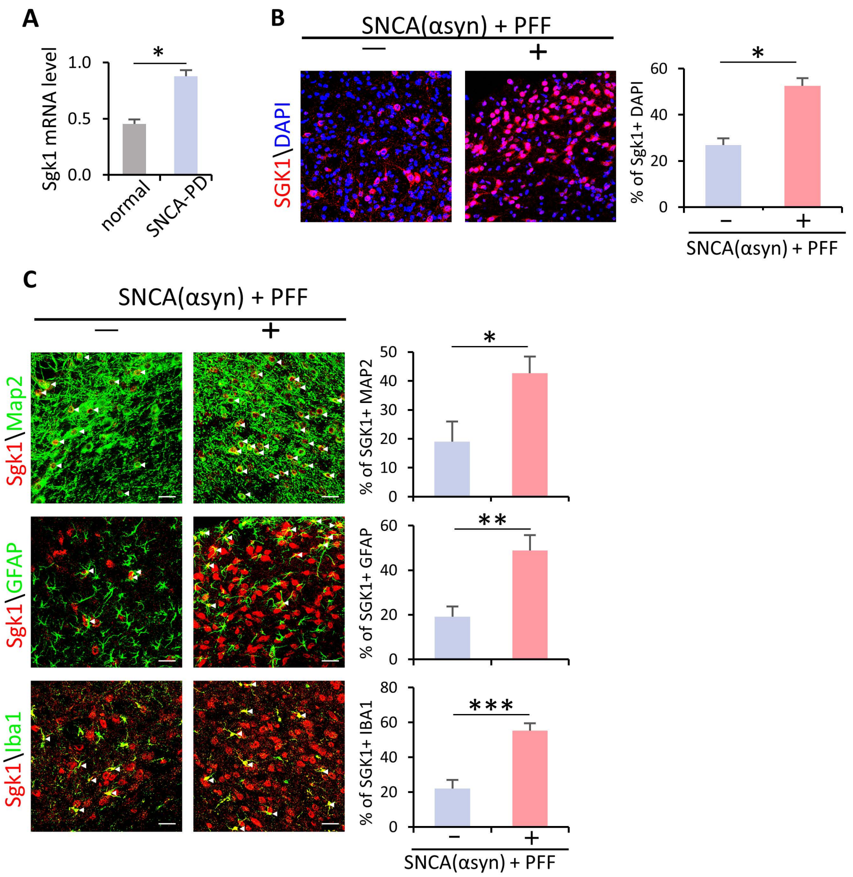

Appendix Figure S9

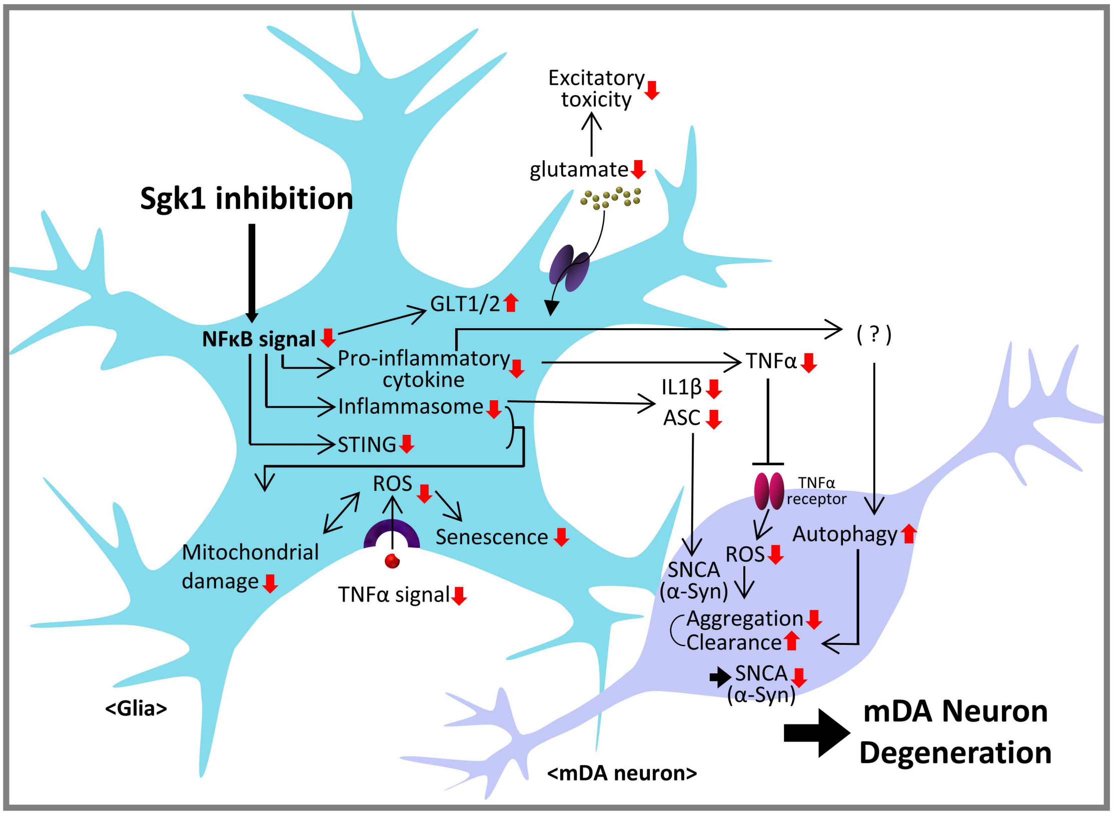

Appendix Table S1. Primer sequences used for qPCR reactions.

Gene	Primer Sequence(Sense/Antisense)	
Casp1	CAC AGC TCT GGA GAT GGT GA	TCT TTC AAG CTT GGG CAC TT
DAI	GCA TTC AAC ATG CAA ACA CC	GGT CGT GTC CTC TAG CAA GC
GLAST	AGT AAG TGG CGG TTT CGG AG	CAGAAGCTCCCCAGGAAAGG
GLT1	ATC CTG GGA GCA GTA TGT GG	CTG ACA GCC CTG TGA TGA GA
iNOS	CAC CTT GGA GTT CAC CCA GT	ACC ACT CGT ACT TGG GAT GC
IFI16	AGG CGG TCA AAC CAA ACA AG	GGG TGA GCC TCC CTT GAA AT
IL-6	TGA AGG ACT CTG GCT TTG TCT	ATG GAT GCT ACC AAA CTG GAT
IL- 1β	CTG TGA CTC GTG GGA TGA TG	GGG ATT TTG TCG TTG CTT GT
IFN-β	TGC CCT CTC CAT CGA CTA CA	GCT GAG GTT GAG CCT TCC AT
NLRP3	ATG CTG CTTCGA CAT CTC CT	AAC CAA TGC GAG ATC CTG AC
PGC1α	ATGTGTCGCCTTCTTGCTCT	ATCTACTGCCTGGGGACCTT
PYCARD(ASC)	ACA GAA GTG GAC GGA GTG CT	CTC CAG GTC CAT CAC CAA GT
SGK1	GTA CCC TGC TCT CGC CTG	ATC TGT ATC CCG ATC CGC CT
TMEM173(STING)	CCC TCT CAA TCT CTC CTG TGC	CTC AGA TCA TCA GGC ACC CC
TNF-α	AGA TGT GGA ACT GGC AGA GG	CCC ATT TGG GAA CTT CTC CT

Appendix Table S2. Primary antibodies used in this study.

Antibodies	Immunostaining	Western Blot	Company
Mouse monoclonal Antibody(Ab)			
ASC	1:500	1:1000	[14]Santa Cruz
Caspase-1(p10)		1:1000	[14]Santa Cruz
Caspase-1(p20)		1:500	[2]AdipoGen
CD16/32	1:500		[3]BD Biosciences
GFAP	1:200		[9]MP Biomedicals
GFP	1:1000		[8]Thermo Fisher Scientific
MAP2	1:1000		[15]Sigma–Aldrich

NeuN	1:100		[10]Millipore
Nitrated αsyn		1:1000	[7]Invitrogen
P-129-αsyn	1:1000		[4]BioLegend
TH	1:200		[6]Immunostar
Tuj1	1:1500		[4]BioLegend
YAP		1:2000	[13]Santa Cruz
α-Synuclein	1:1000	1:2000	[2]BD Biosciences
ß-actin		1:2000	[7]Invitrogen
Rabbit polyclonal Ab			
cGAS		1:2000	[5]Cell signaling
HMGB1		1:1000	[5]Cell signaling
GFP	1:1000		[8]Thermo Fisher Scientific
Iba-1	1:200		[14]Wako
IKKα/ß		1:1500	[5]Cell signaling
IL-6		1:1000	[5]Cell signaling
IκBα		1:2000	[5]Cell signaling
IRF3		1:2000	[5]Cell signaling
Lamin B1		1:1000	[5]Cell signaling
LATS1		1:1000	[5]Cell signaling
LATS2		1:1000	[5]Cell signaling
MMP3		1:1000	[5]Cell signaling
MST1		1:1000	[5]Cell signaling
MST2		1:1000	[5]Cell signaling
PGC1α		1:1000	[1]Abcam
P16 INK4A		1:1000	[5]Cell signaling
p65 (NFkB)		1:1000	[5]Cell signaling
p-IKKα/ß		1:1000	[5]Cell signaling
p-IκBα		1:2000	[5]Cell signaling
p-p65(p-NFkB)		1:1500	[5]Cell signaling
p-TBK1		1:1000	[5]Cell signaling

p-IRF3		1:2000	[5]Cell signaling
p-s127-YAP		1:2000	[5]Cell signaling
p-s397-YAP		1:2000	[5]Cell signaling
SGK1		1:1000	[10]Millipore
SGK1		1:500	[1]Abcam
STING		1:1000	[1]Abcam
TH	1:1000		[11]Pel-freez
TBK1		1:1000	[5]Cell signaling
Tuj1	1:1500		[4]BioLegend
TNFα		1:1000	[5]Cell signaling
YAP/TAZ		1:1000	[5]Cell signaling
Rat polyclonal Ab			
DAT	1:200		[1]Abcam
Goat polyclonal Ab			
IL-1β		1:2000	[12]R&D Systems
TNFα		1:2000	[12]R&D Systems

1. Abcam, Cambridge, MA, UK
2. AdipoGen, San Diego, CA, USA
3. BD Biosciences, Franklin Lakes, NJ, USA
4. BioLegend, San Diego, CA, USA
5. Cell signaling, Danvers, MA, USA
6. Immunostar, Hudson, WI, USA
7. Invitrogen, Carlsbad, CA, USA
8. Thermo Fisher Scientific, Waltham, MA, USA
9. MP Biomedicals, Santa Ana, CA, USA
10. Millipore, Pittsburgh, PA, USA
11. Pel-Freez, Rogers, AR, USA
12. R&D Systems, Minneapolis, MN, USA
13. Santa Cruz Biotechnology, Santa Cruz, CA, USA
14. Wako, Osaka, Japan
15. Sigma–Aldrich, Saint Louis, MO, USA
