# Supplementary material for: Author Correction: SGK1 inhibition in glia ameliorates pathologies and symptoms in Parkinson disease animal models
Source: EMBO Mol Med. 2025 Aug 11;17(9):2525–9. doi: 10.1038/s44321-025-00270-y (PMC12423325; doi:10.1038/s44321-025-00270-y)
Supplement: Supplementary file 1 — Source Data for corrected figures [file 44321_2025_270_MOESM1_ESM.zip › (corri_emmm202013076) Data Integrity Off-Res Metadata/Fig 7T & 9D.pptx]

## Slide 1
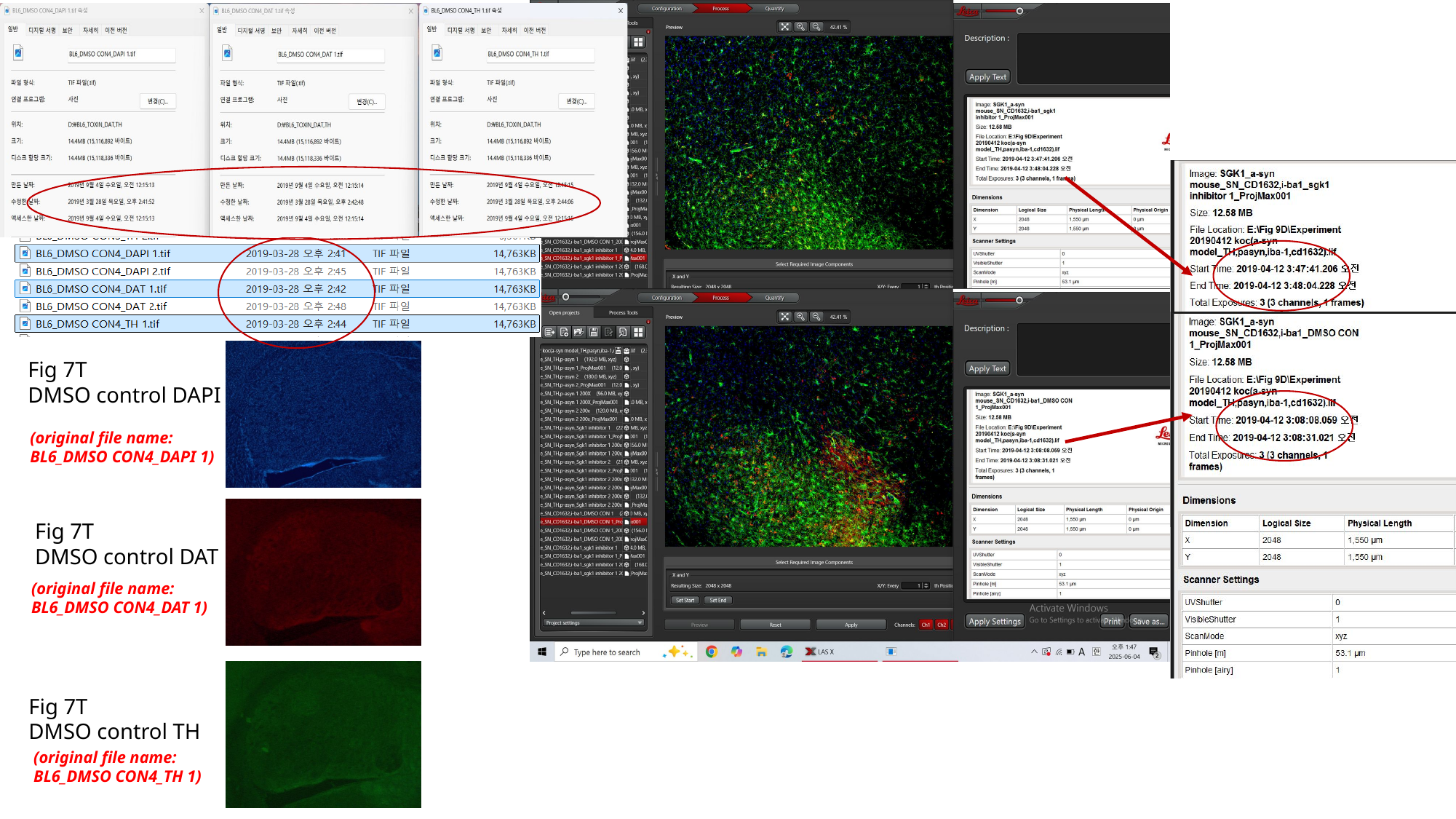

Fig 7T
DMSO control DAPI
(original file name:
BL6_DMSO CON4_DAPI 1)
Fig 7T
DMSO control DAT
(original file name:
BL6_DMSO CON4_DAT 1)
Fig 7T
DMSO control TH
(original file name:
BL6_DMSO CON4_TH 1)
