# Supplementary figures and images for: Author Correction: SGK1 inhibition in glia ameliorates pathologies and symptoms in Parkinson disease animal models
Source: EMBO Mol Med. 2025 Aug 11;17(9):2525–9. doi: 10.1038/s44321-025-00270-y (PMC12423325; doi:10.1038/s44321-025-00270-y)

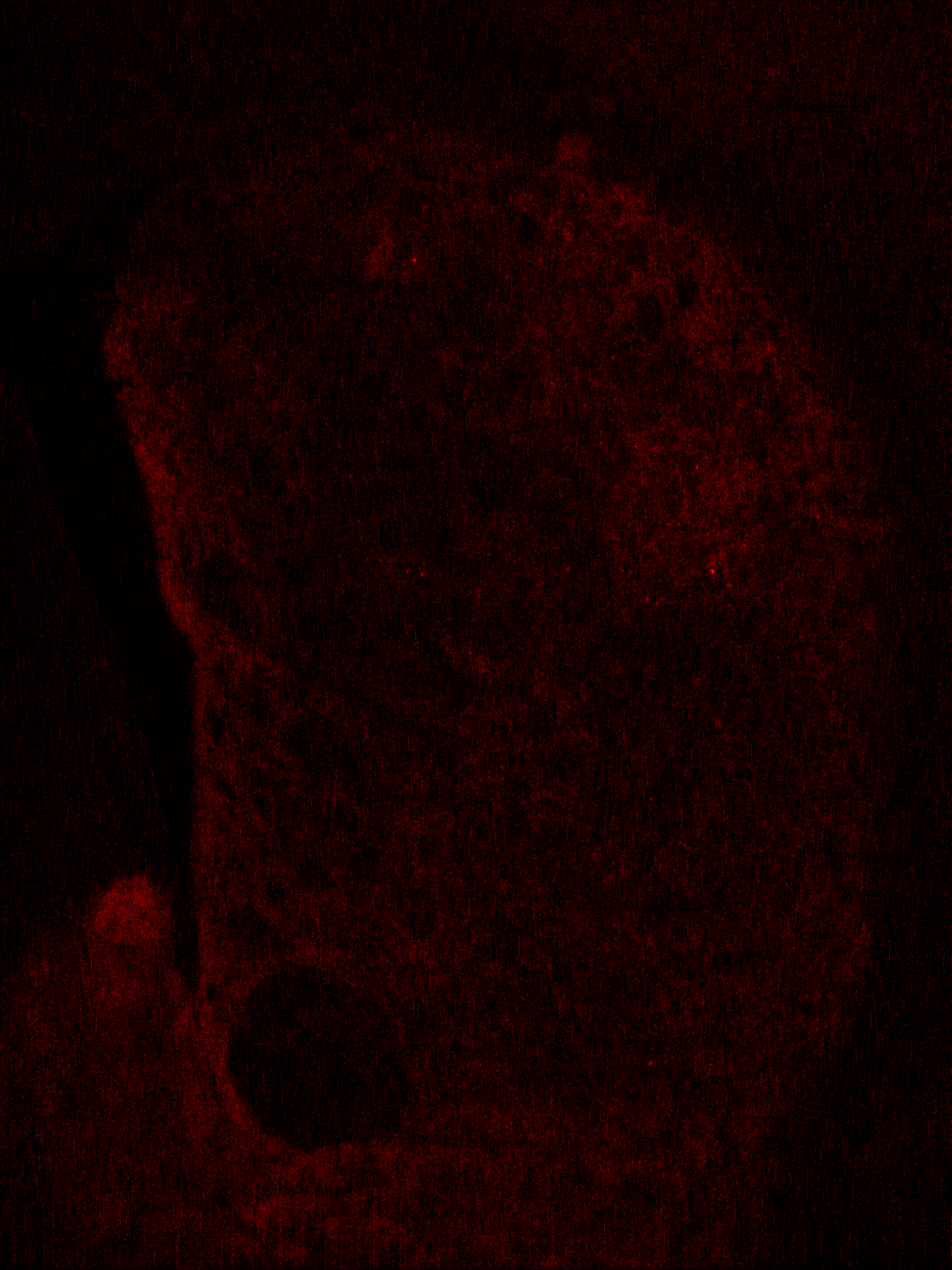

Supplement: Supplementary file 1 — Source Data for corrected figures [file 44321_2025_270_MOESM1_ESM.zip › EMM-2024-21123-Source_data_files_Figure_7T_8N_9D-sd/Figure7T_(DMSO Con)_DAT.tif]

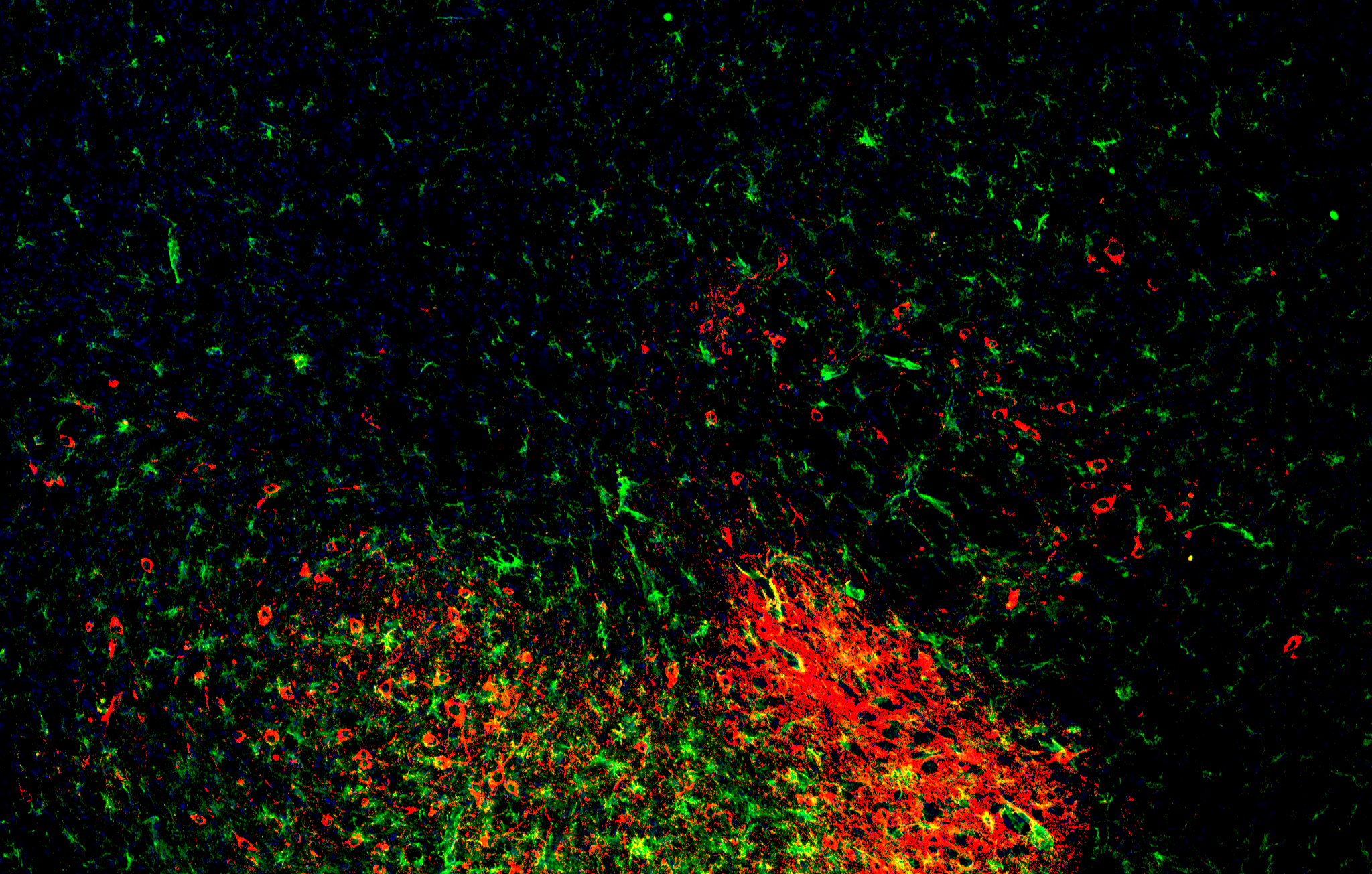

Supplement: Supplementary file 1 — Source Data for corrected figures [file 44321_2025_270_MOESM1_ESM.zip › EMM-2024-21123-Source_data_files_Figure_7T_8N_9D-sd/Figure9D_(SN_CD1632, iba1_DMSO CON).tif]

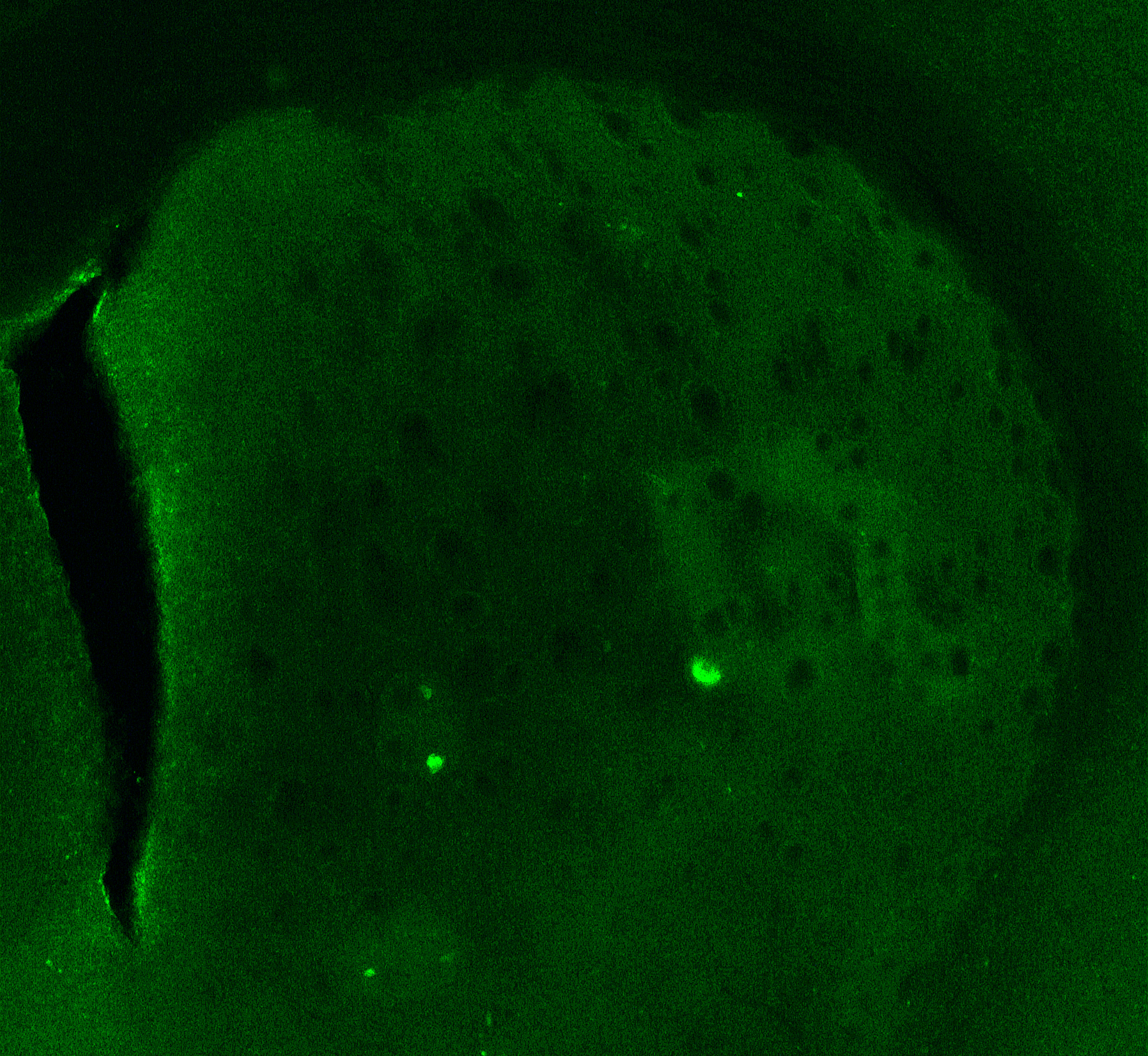

Supplement: Supplementary file 1 — Source Data for corrected figures [file 44321_2025_270_MOESM1_ESM.zip › EMM-2024-21123-Source_data_files_Figure_7T_8N_9D-sd/Figure8N_(ShCon)_TH.tif]

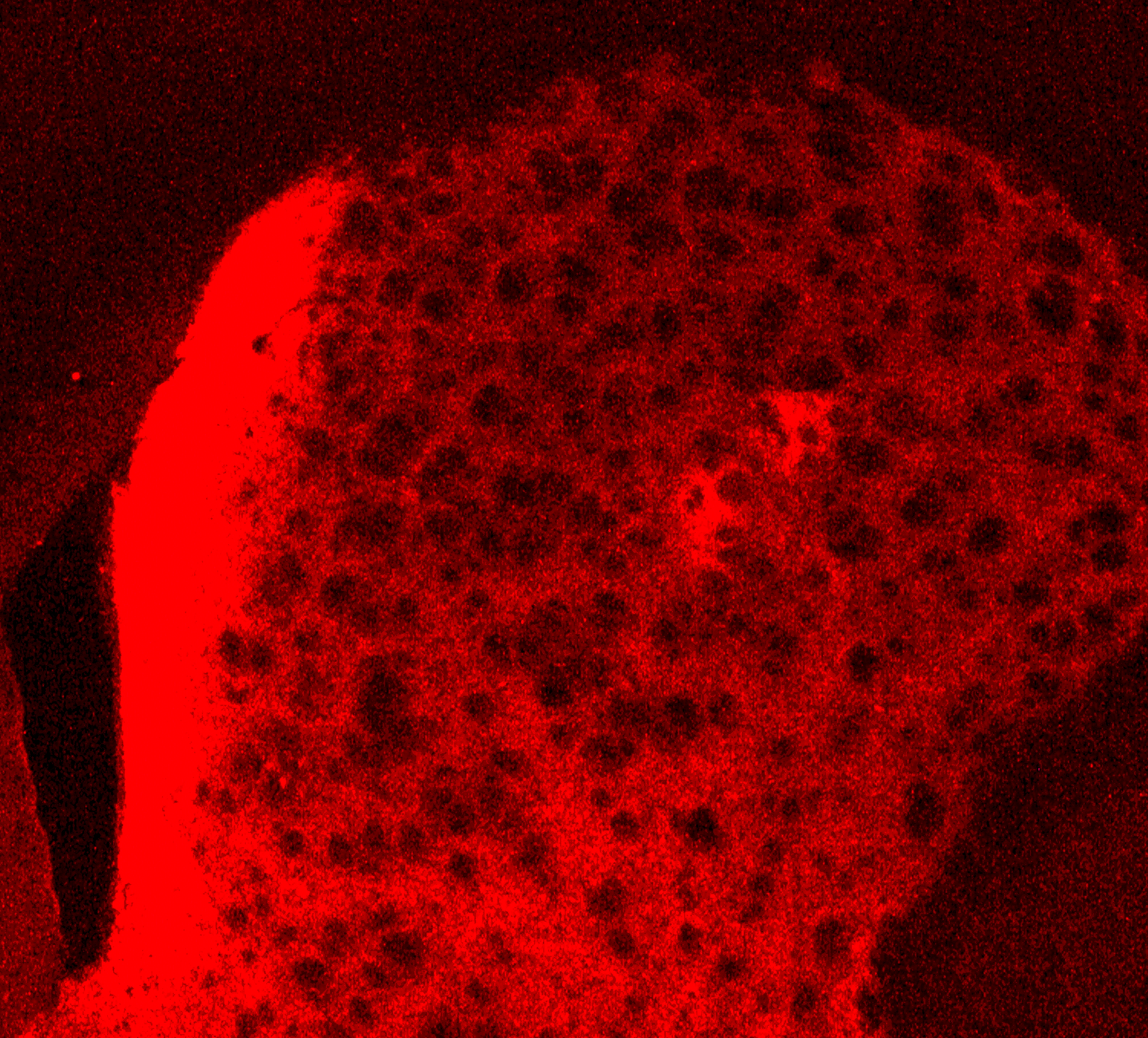

Supplement: Supplementary file 1 — Source Data for corrected figures [file 44321_2025_270_MOESM1_ESM.zip › EMM-2024-21123-Source_data_files_Figure_7T_8N_9D-sd/Figure8N_(Shsgk1)_DAT.tif]

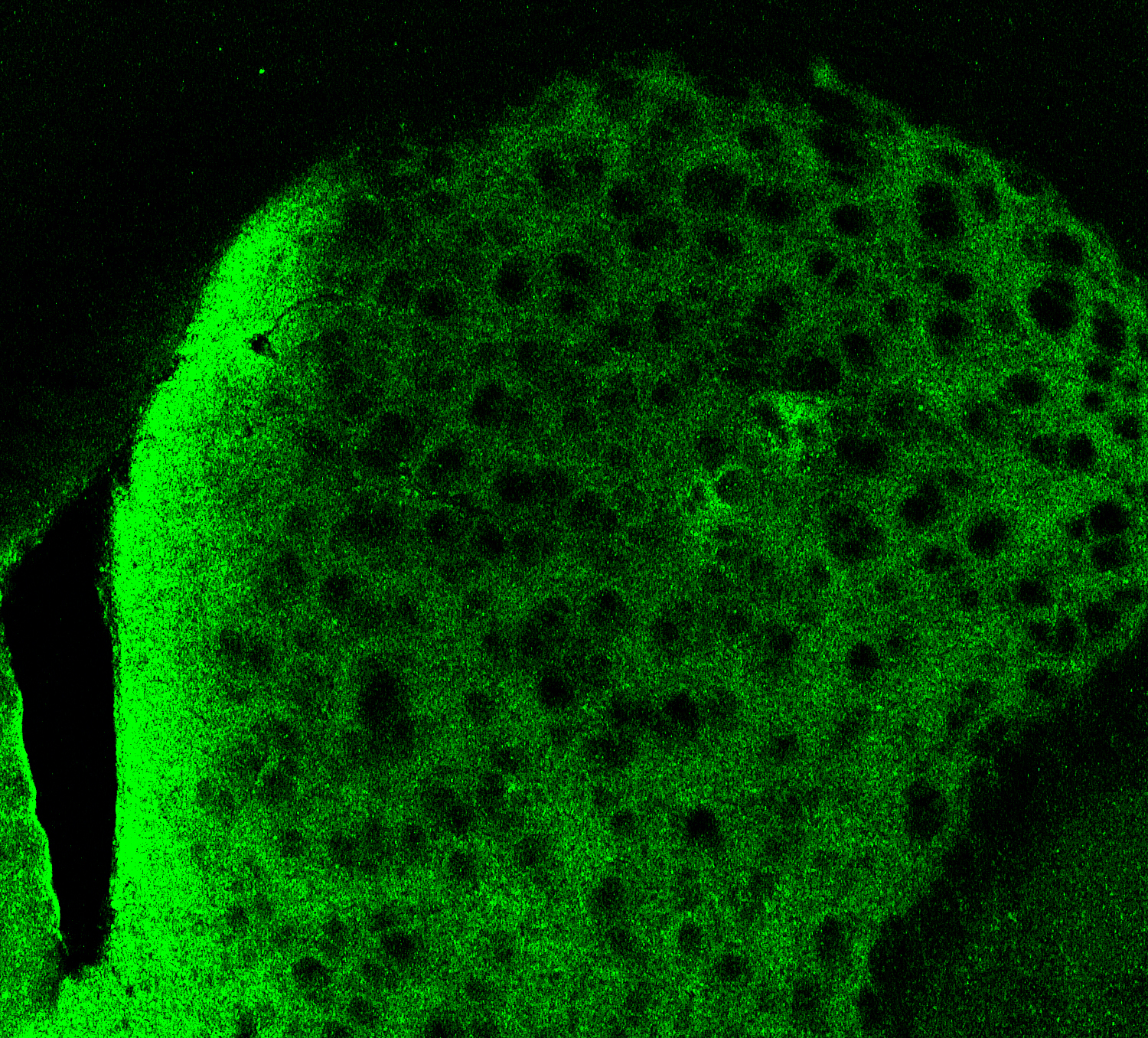

Supplement: Supplementary file 1 — Source Data for corrected figures [file 44321_2025_270_MOESM1_ESM.zip › EMM-2024-21123-Source_data_files_Figure_7T_8N_9D-sd/Figure8N_(Shsgk1)_TH.tif]

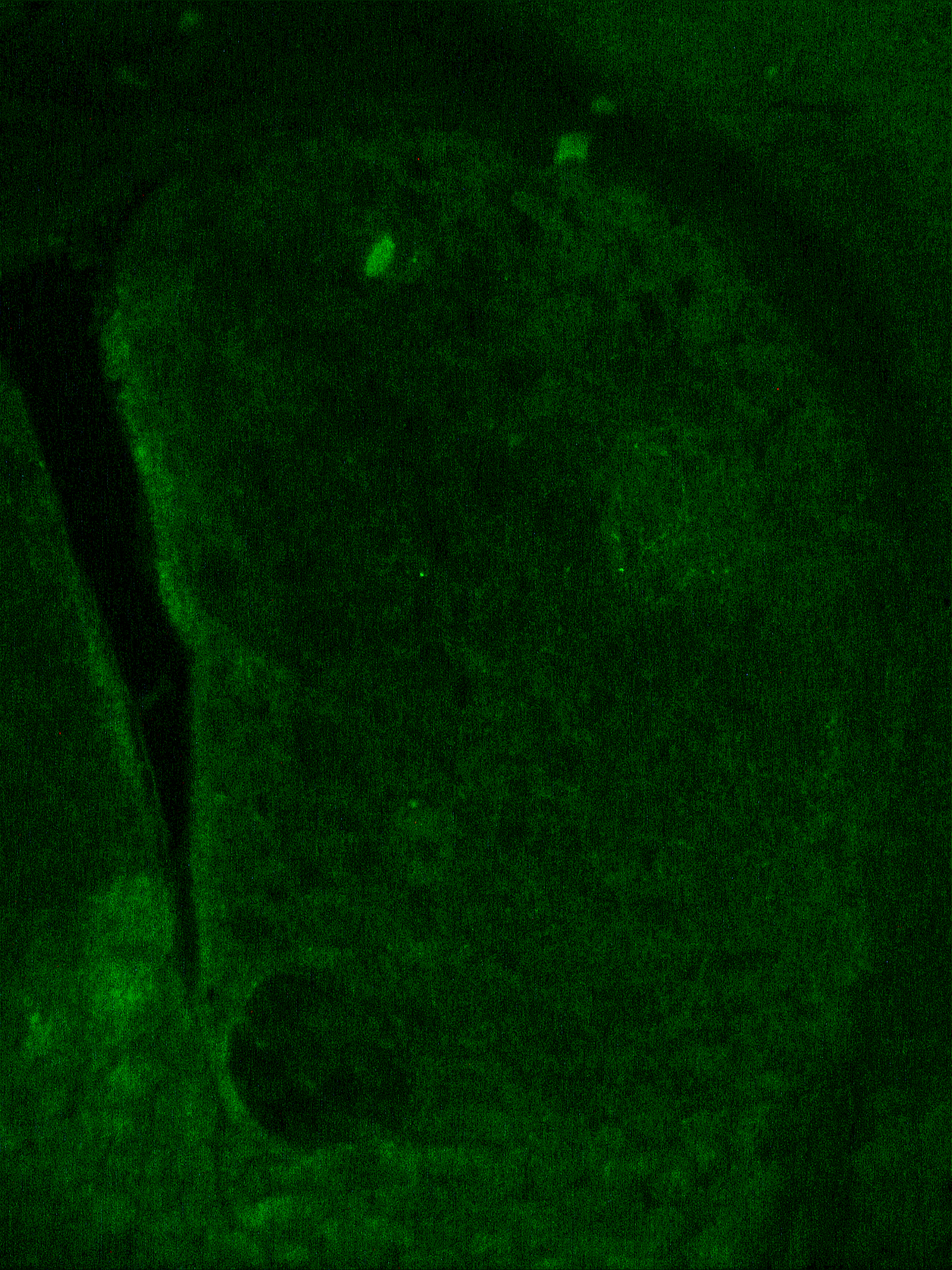

Supplement: Supplementary file 1 — Source Data for corrected figures [file 44321_2025_270_MOESM1_ESM.zip › EMM-2024-21123-Source_data_files_Figure_7T_8N_9D-sd/Figure7T_(DMSO Con).tif]

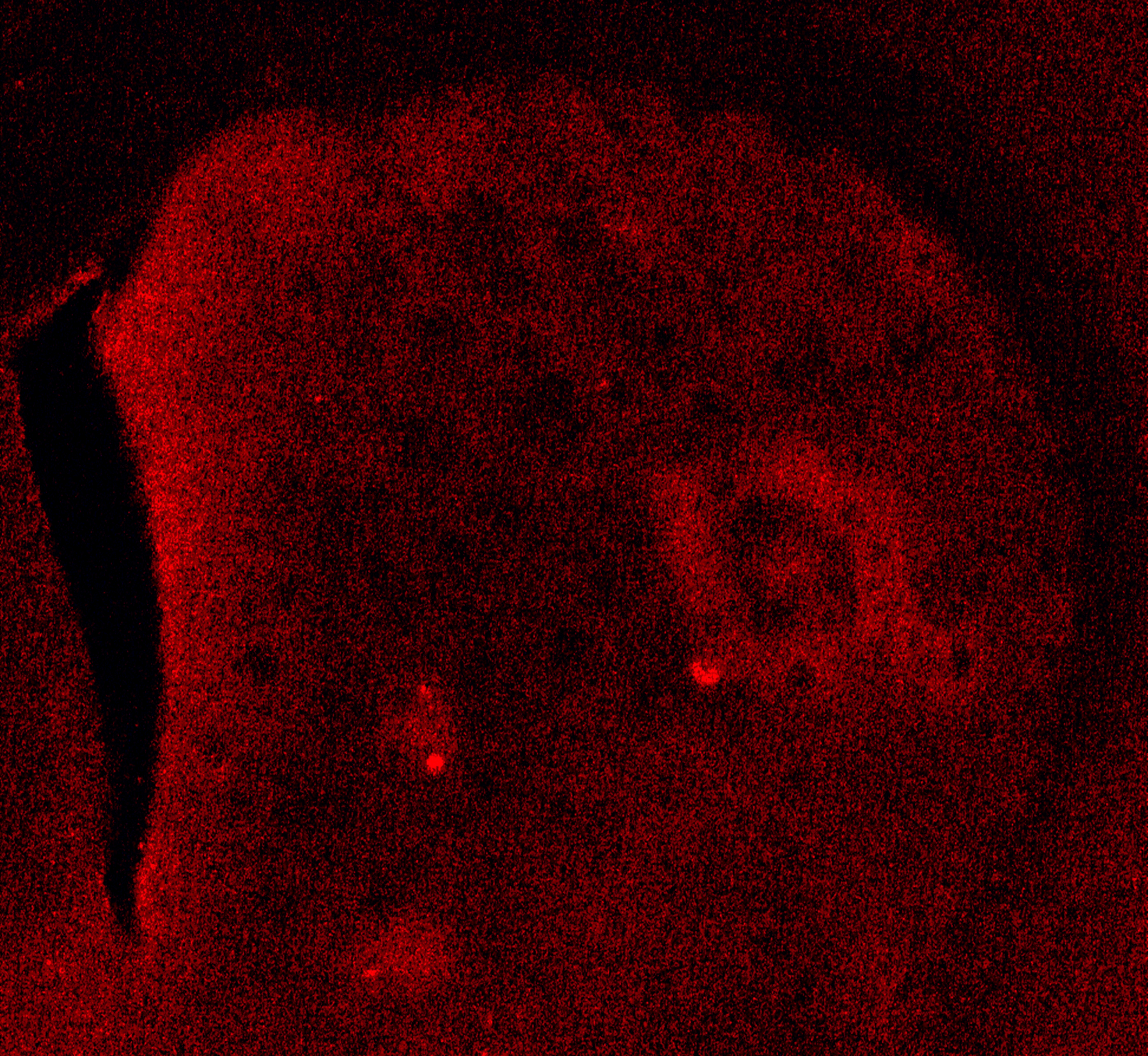

Supplement: Supplementary file 1 — Source Data for corrected figures [file 44321_2025_270_MOESM1_ESM.zip › EMM-2024-21123-Source_data_files_Figure_7T_8N_9D-sd/Figure8N_(ShCon)_DAT.tif]

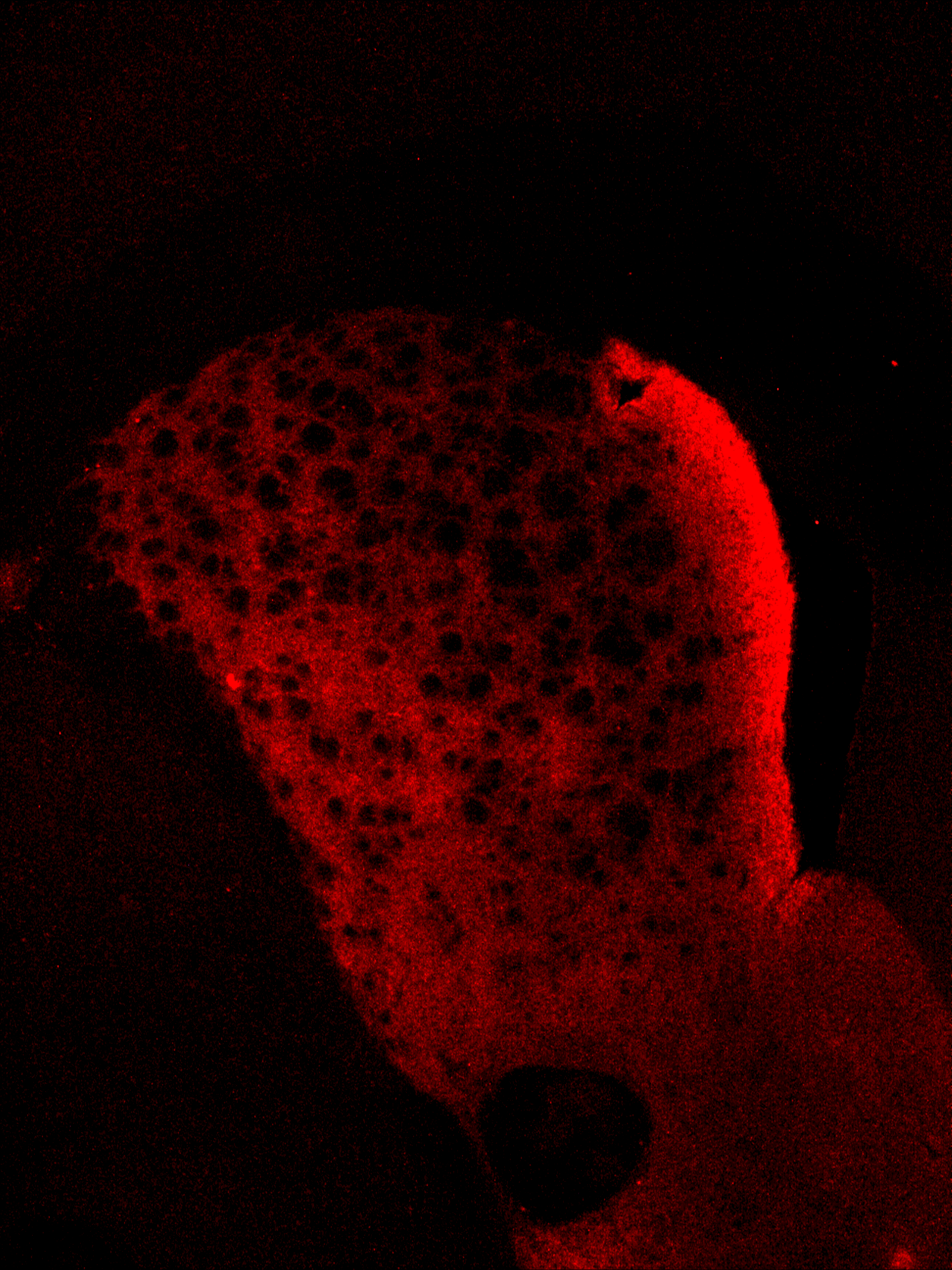

Supplement: Supplementary file 1 — Source Data for corrected figures [file 44321_2025_270_MOESM1_ESM.zip › EMM-2024-21123-Source_data_files_Figure_7T_8N_9D-sd/Figure7T_(sgk1 in)_DAT.tif]

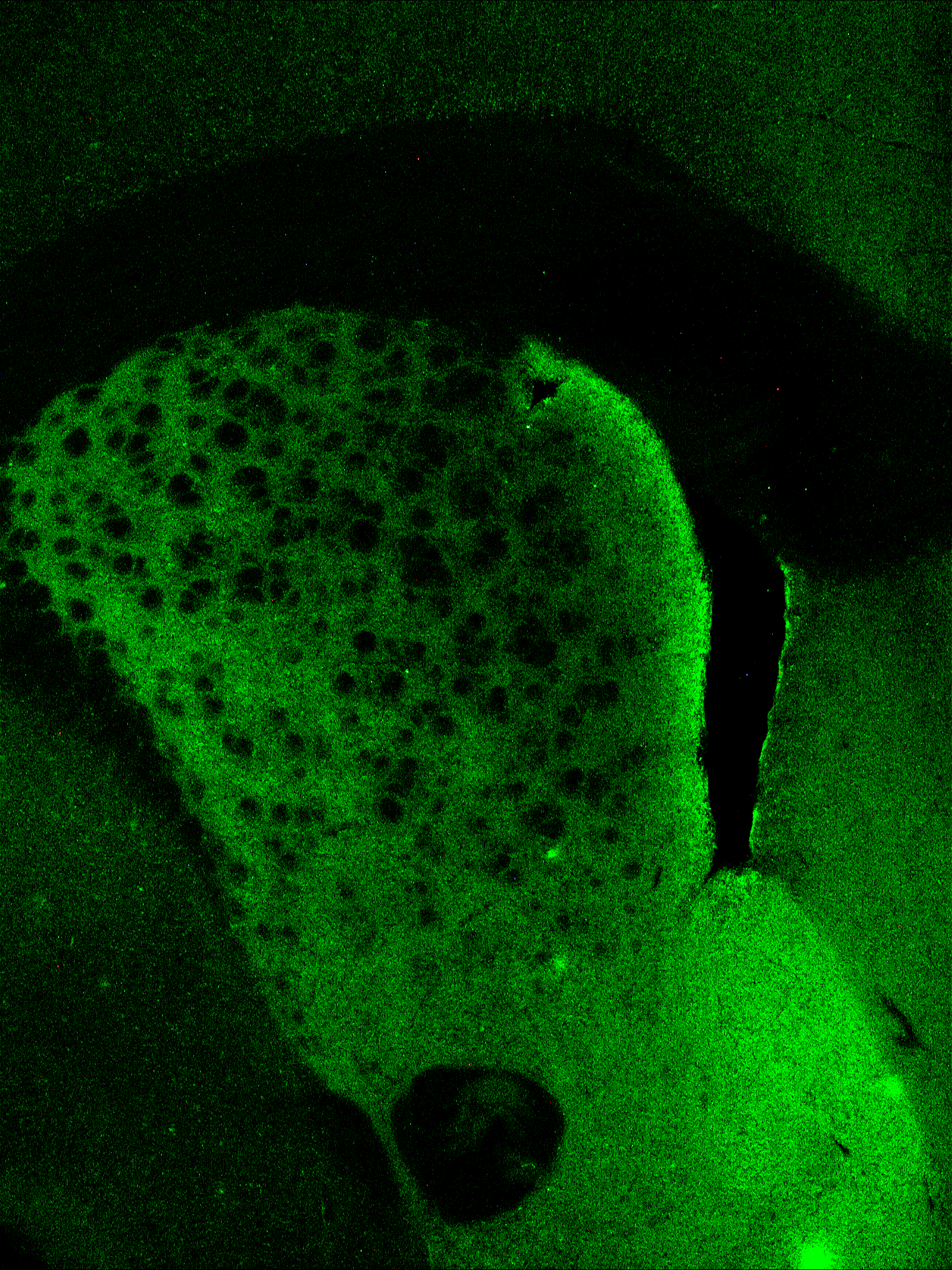

Supplement: Supplementary file 1 — Source Data for corrected figures [file 44321_2025_270_MOESM1_ESM.zip › EMM-2024-21123-Source_data_files_Figure_7T_8N_9D-sd/Figure7T_(sgk1 in)_TH.tif]

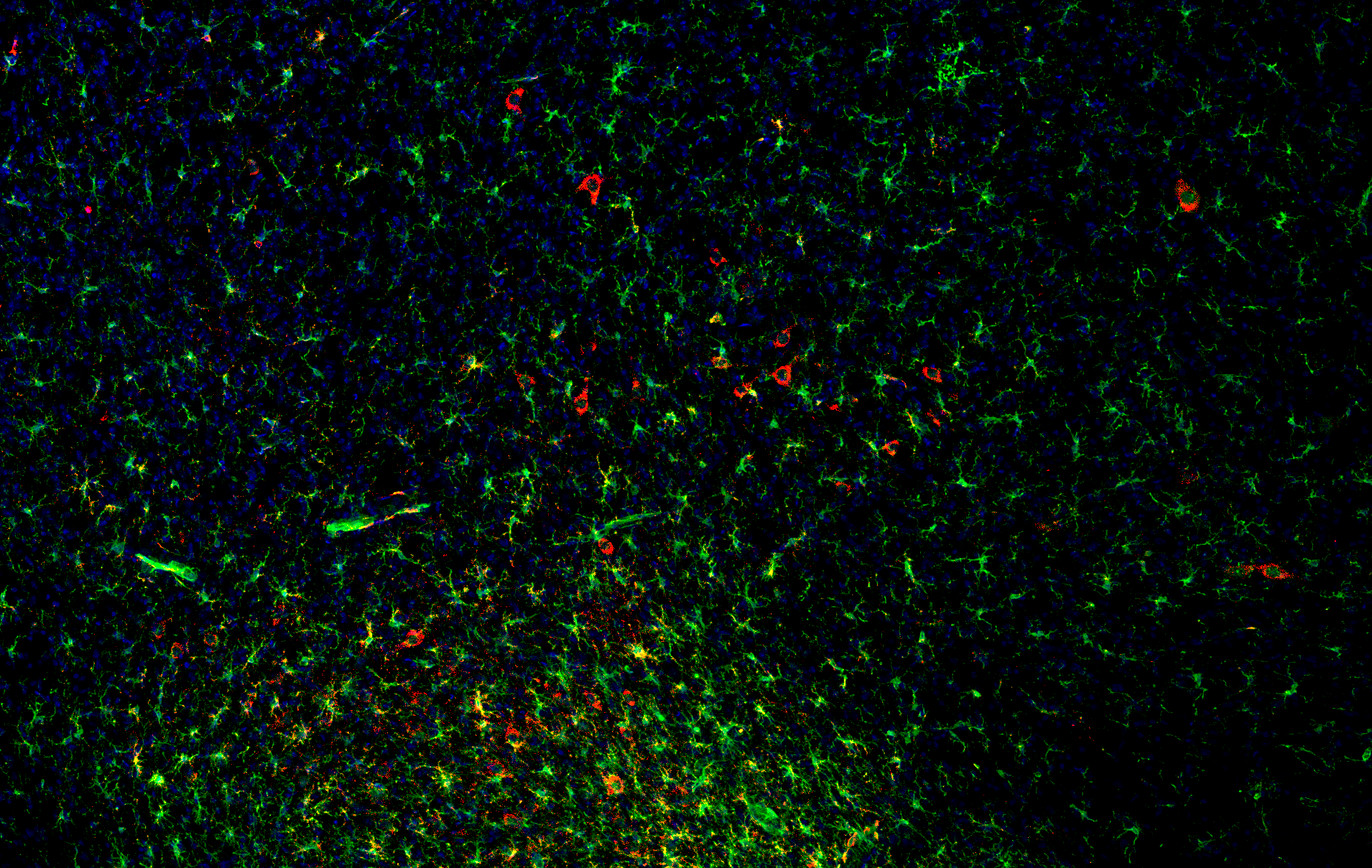

Supplement: Supplementary file 1 — Source Data for corrected figures [file 44321_2025_270_MOESM1_ESM.zip › EMM-2024-21123-Source_data_files_Figure_7T_8N_9D-sd/Figure9D_(SN_CD1632, iba1_sgk1 inhibitor).tif]

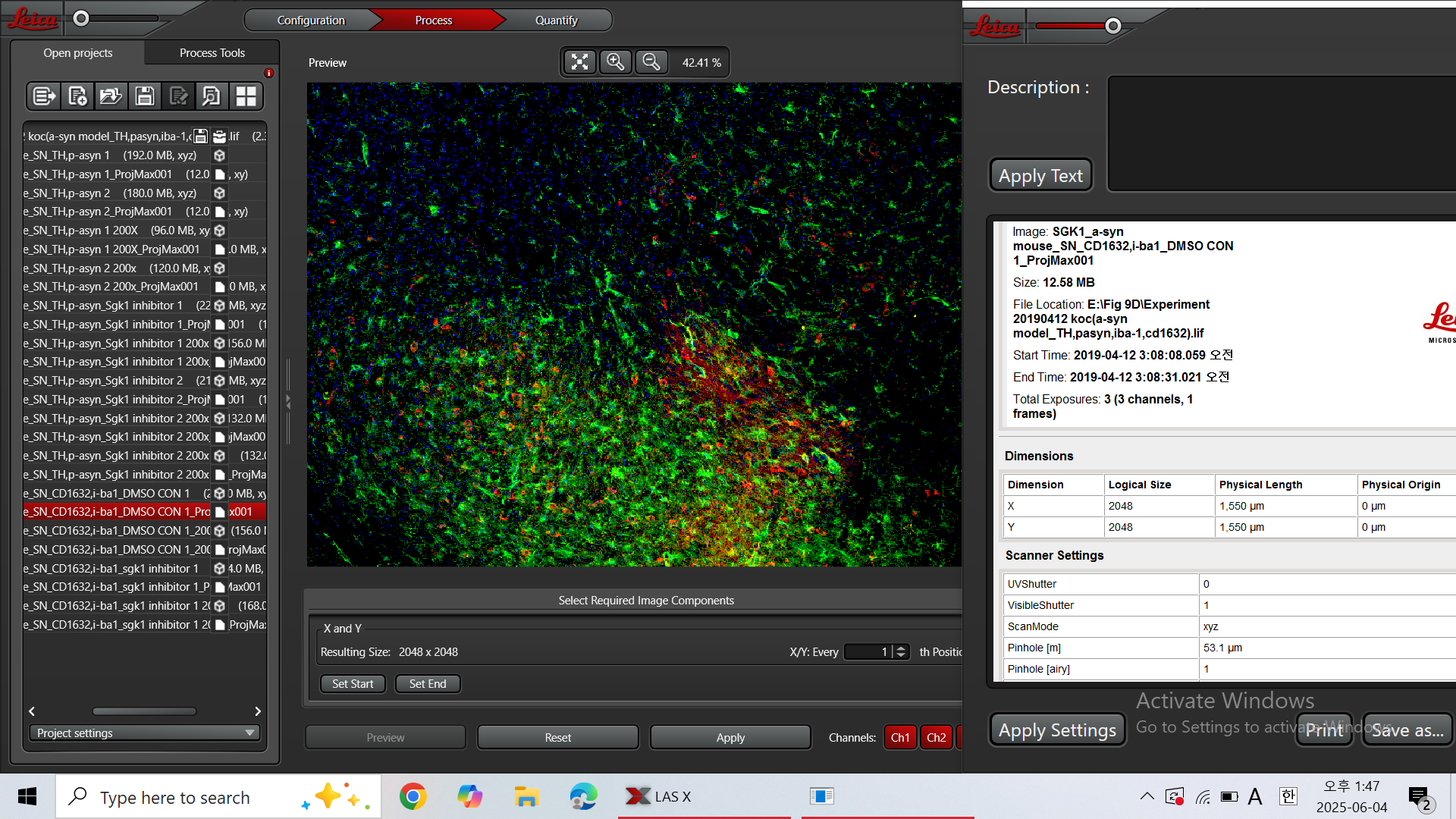

Supplement: Supplementary file 1 — Source Data for corrected figures [file 44321_2025_270_MOESM1_ESM.zip › (corri_emmm202013076) Data Integrity Off-Res Metadata/FIG 9- IMG_4523.png]

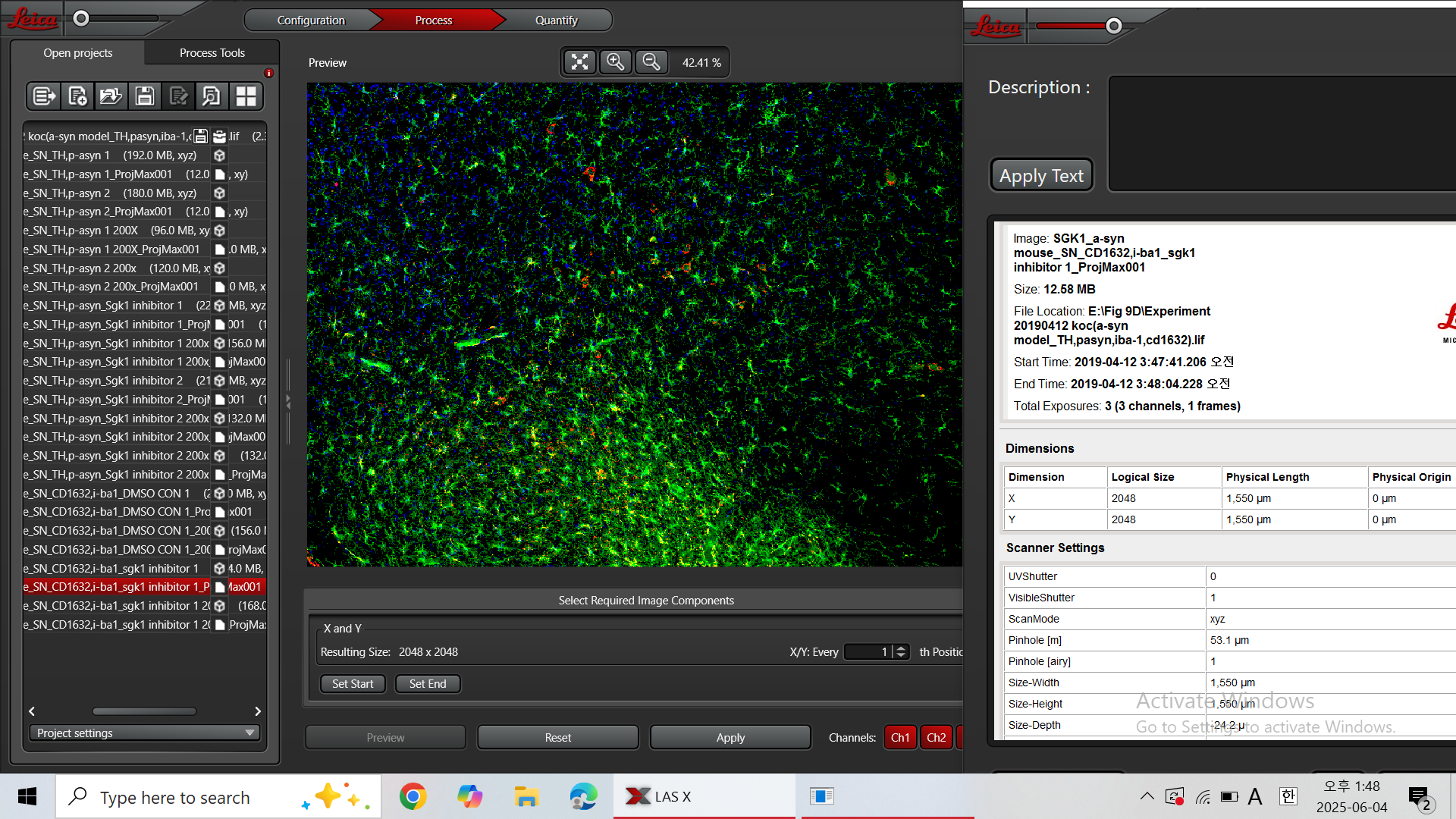

Supplement: Supplementary file 1 — Source Data for corrected figures [file 44321_2025_270_MOESM1_ESM.zip › (corri_emmm202013076) Data Integrity Off-Res Metadata/FIG 9- IMG_4522.png]

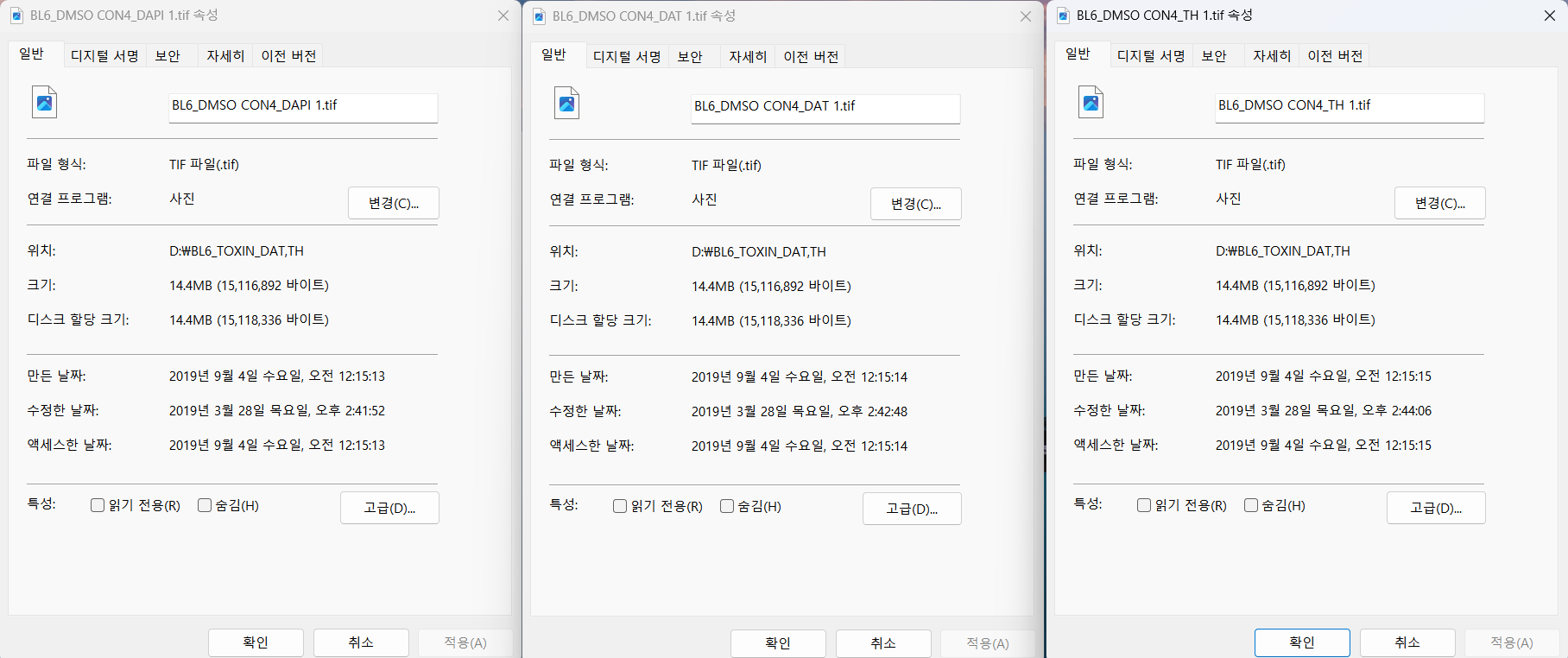

Supplement: Supplementary file 1 — Source Data for corrected figures [file 44321_2025_270_MOESM1_ESM.zip › (corri_emmm202013076) Data Integrity Off-Res Metadata/FIG 7- a-syn model_DMSO CON_Screenshot_2.png]

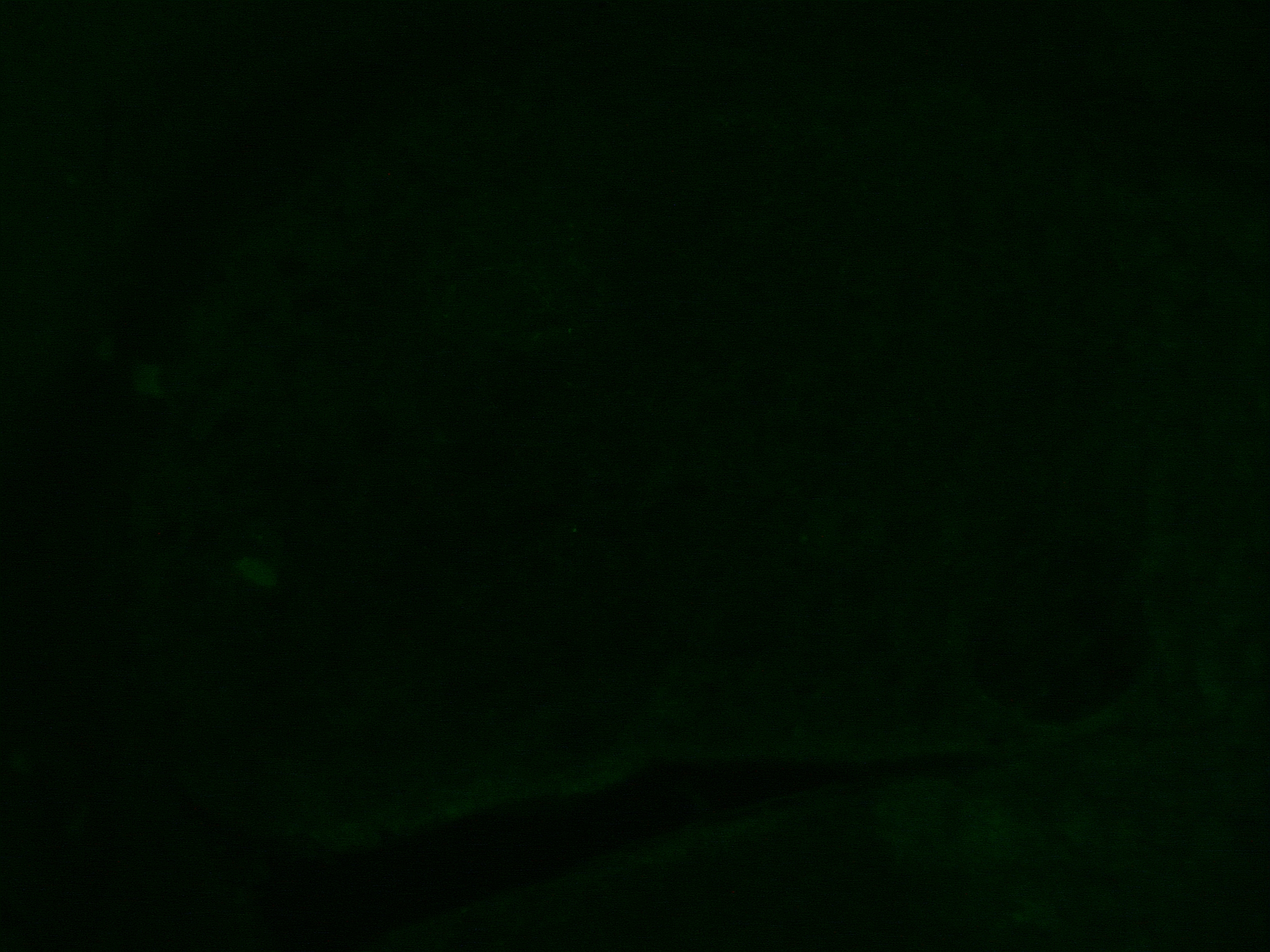

Supplement: Supplementary file 1 — Source Data for corrected figures [file 44321_2025_270_MOESM1_ESM.zip › (corri_emmm202013076) Data Integrity Off-Res Metadata/FIG 7- BL6_DMSO CON4_TH 1.tif]

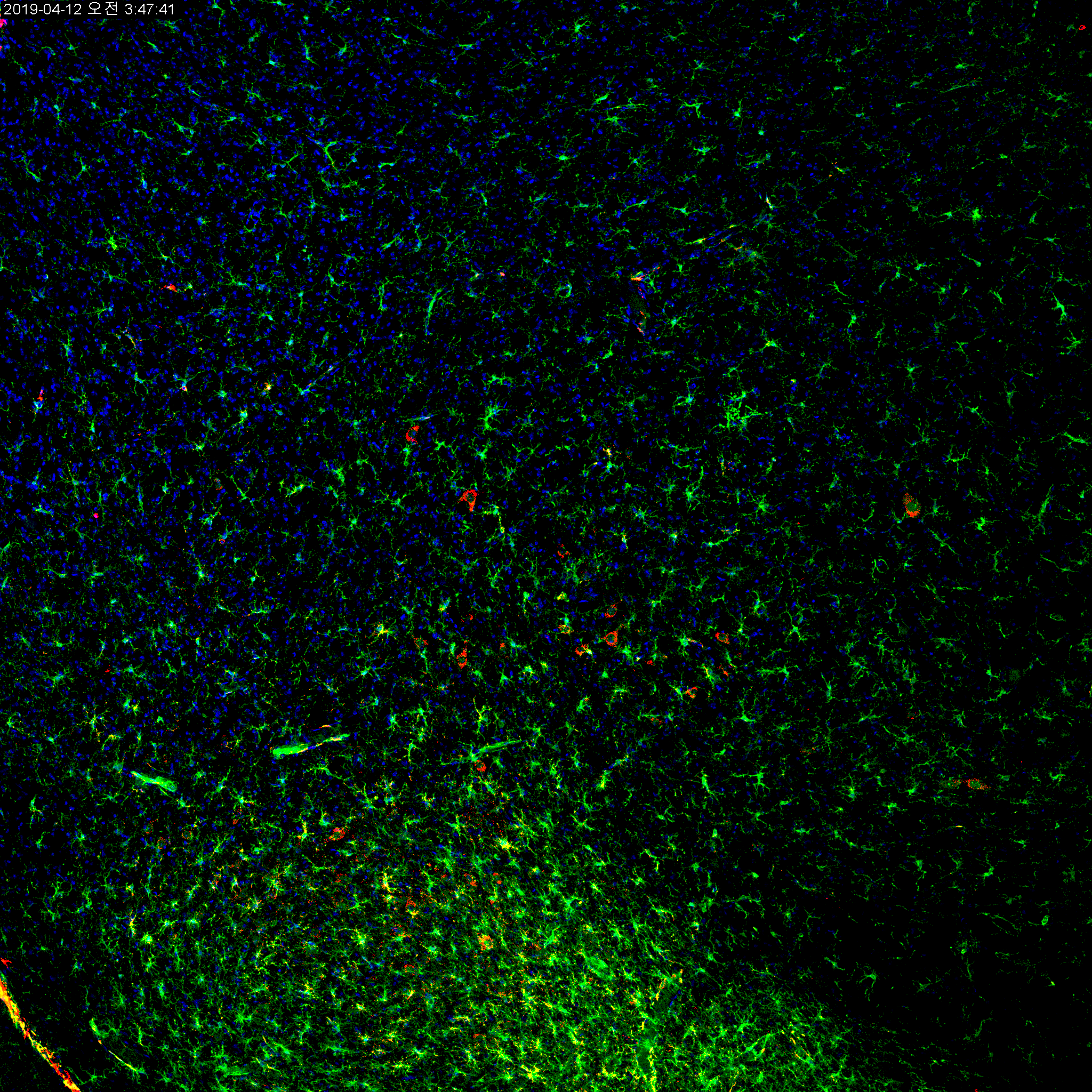

Supplement: Supplementary file 1 — Source Data for corrected figures [file 44321_2025_270_MOESM1_ESM.zip › (corri_emmm202013076) Data Integrity Off-Res Metadata/FIG 9- SGK1_a-syn mouse_SN_CD1632,i-ba1_sgk1 inhibitor 1_ProjMax001 (1).tif]

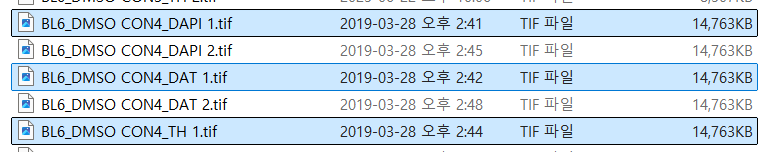

Supplement: Supplementary file 1 — Source Data for corrected figures [file 44321_2025_270_MOESM1_ESM.zip › (corri_emmm202013076) Data Integrity Off-Res Metadata/FIG7-a-syn model_DMSO CON_Screenshot_1.png]

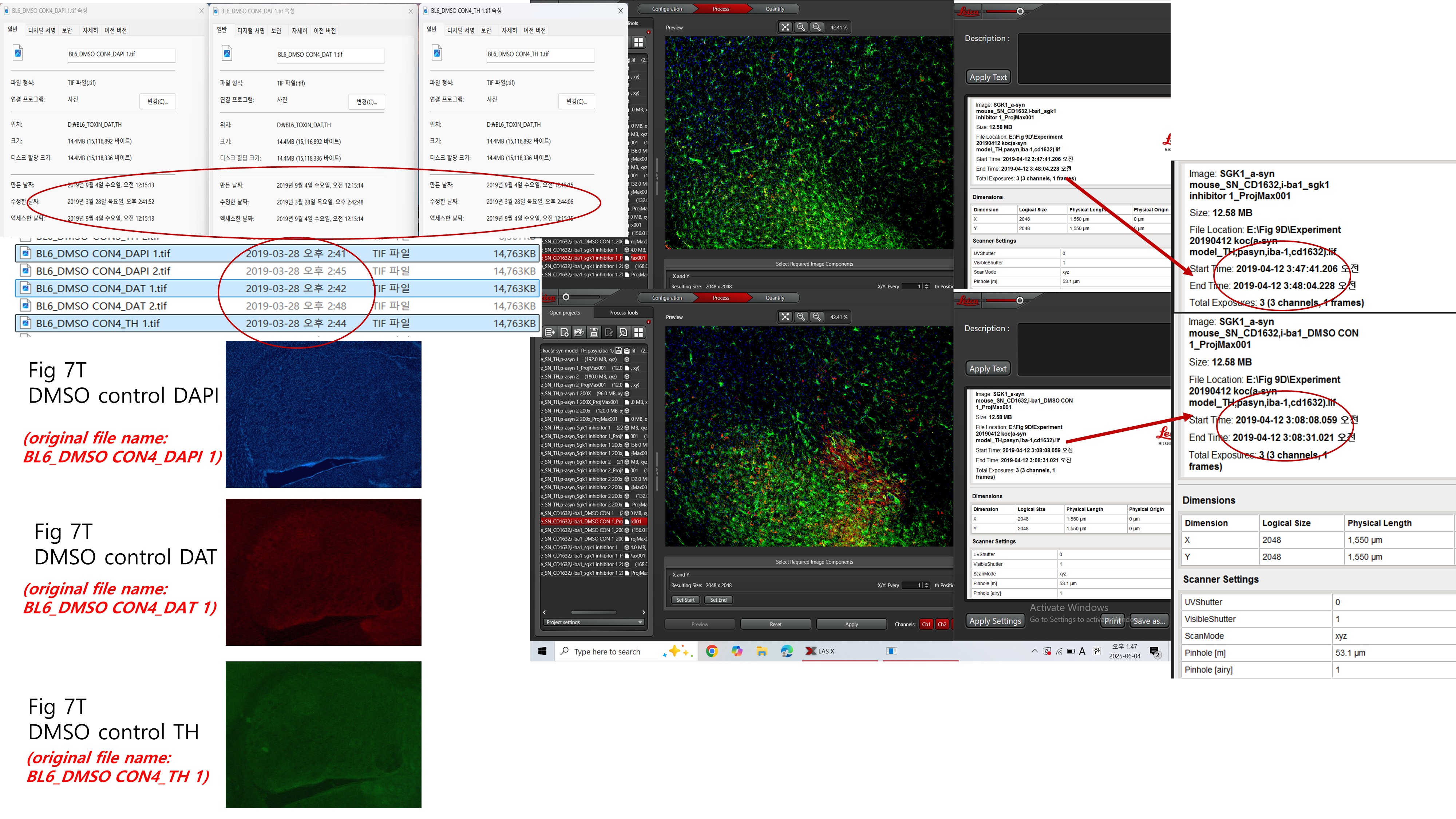

Supplement: Supplementary file 1 — Source Data for corrected figures [file 44321_2025_270_MOESM1_ESM.zip › (corri_emmm202013076) Data Integrity Off-Res Metadata/Fig 7T & 9D.jpg]

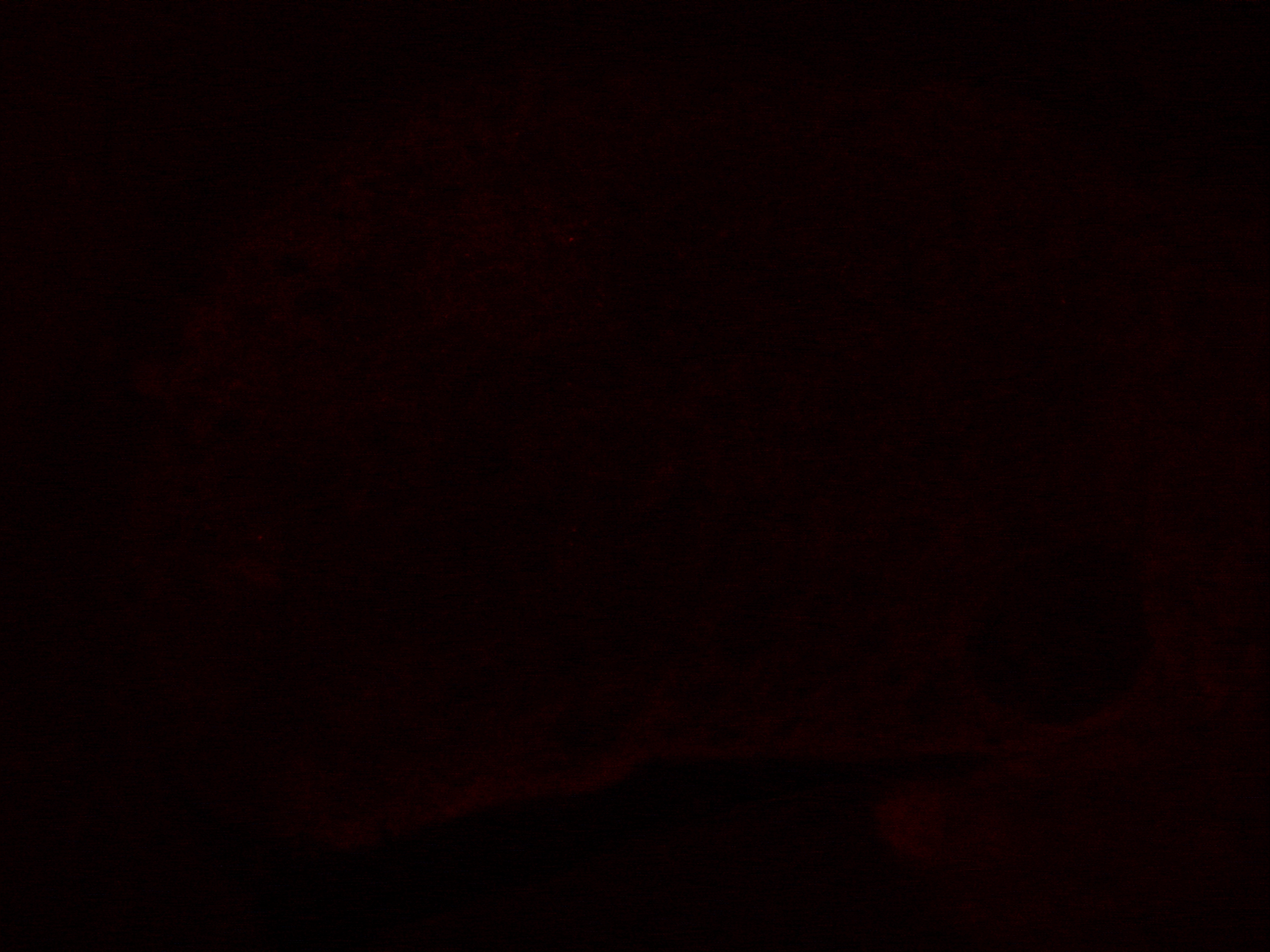

Supplement: Supplementary file 1 — Source Data for corrected figures [file 44321_2025_270_MOESM1_ESM.zip › (corri_emmm202013076) Data Integrity Off-Res Metadata/FIG 7- BL6_DMSO CON4_DAT 1.tif]

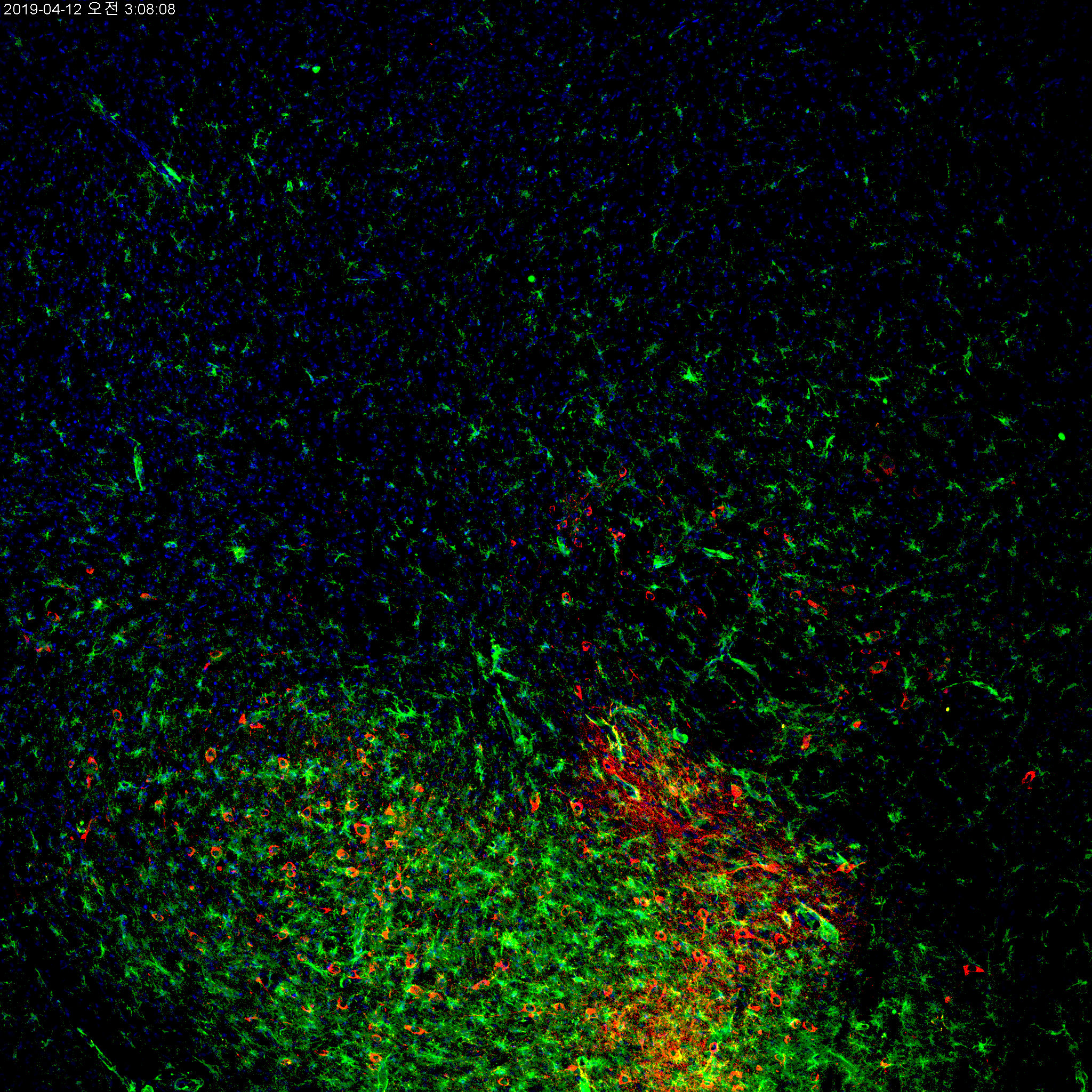

Supplement: Supplementary file 1 — Source Data for corrected figures [file 44321_2025_270_MOESM1_ESM.zip › (corri_emmm202013076) Data Integrity Off-Res Metadata/FIG 9- SGK1_a-syn mouse_SN_CD1632,i-ba1_DMSO CON 1_ProjMax001 (1).tif]

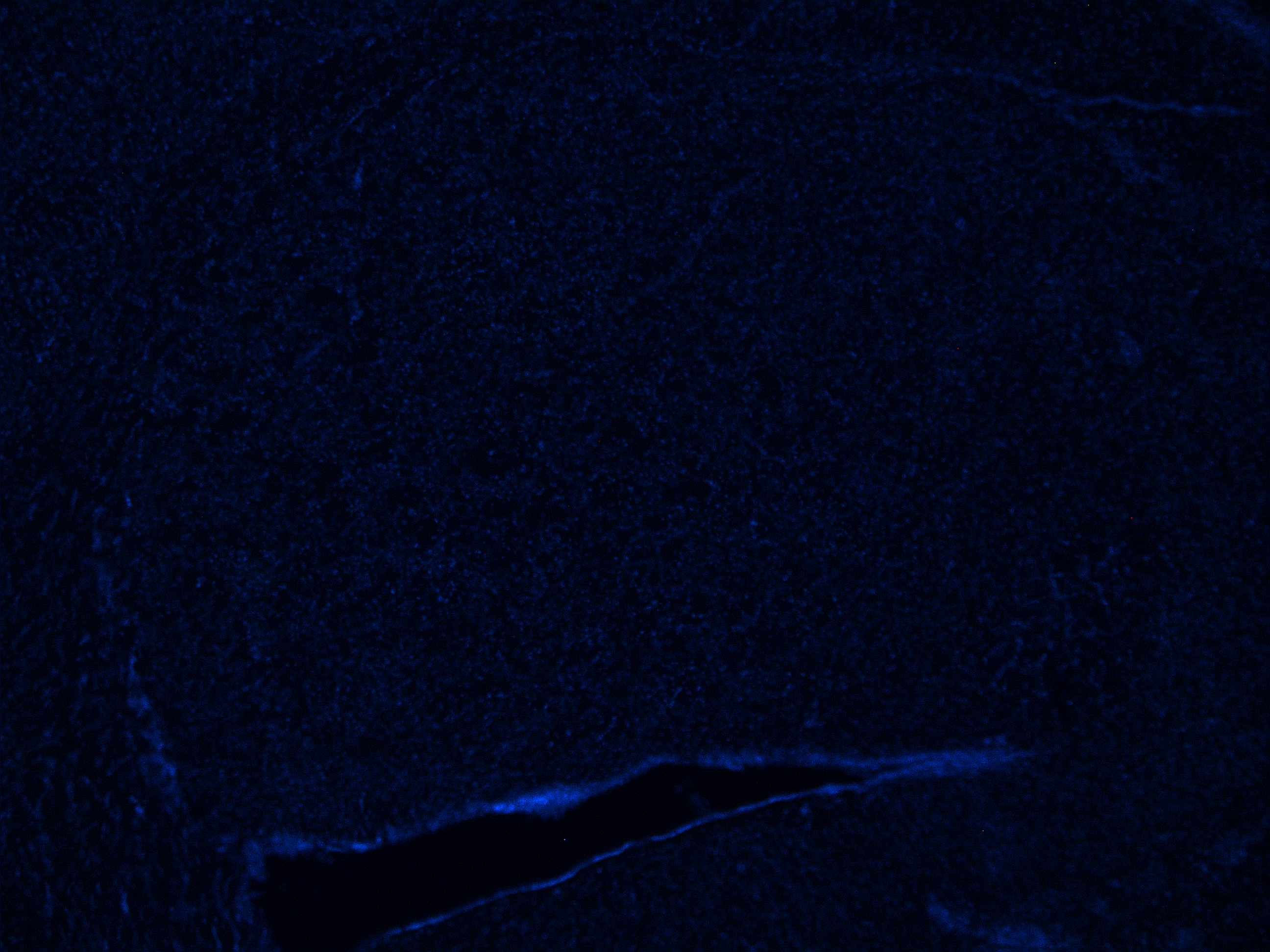

Supplement: Supplementary file 1 — Source Data for corrected figures [file 44321_2025_270_MOESM1_ESM.zip › (corri_emmm202013076) Data Integrity Off-Res Metadata/FIG 7- BL6_DMSO CON4_DAPI 1.tif]
